# Long COVID Does Not Impair Hemodynamic, Vascular, or Autonomic Responses to Maximal Exercise: Sex-Stratified Study in Young Adults

**DOI:** 10.3390/jpm16010038

**Published:** 2026-01-07

**Authors:** Carla Nascimento dos Santos Rodrigues, Fernanda Rico Angelotto, Vitória Luiz Diotto, Daniel da Motta Cristofoletti, Tatiana Oliveira Passos de Araújo, Marco Antonio de Lima, José Campanholi Neto, Jonato Prestes, James Navalta, Guilherme Borges Pereira

**Affiliations:** 1 Laboratory of Clinical Exercise Physiology, Department of Physiological Sciences, Federal University of São Carlos, São Carlos 13565-905, Brazil; crodrigues@estudante.ufscar.br (C.N.d.S.R.); fernandara@estudante.ufscar.br (F.R.A.); vitludiotto@gmail.com (V.L.D.); danielmc@estudante.ufscar.br (D.d.M.C.); tatiana.passos@ufscar.br (T.O.P.d.A.); malima@estudante.ufscar.br (M.A.d.L.); 2 Department of Morphology and Pathology, Federal University of São Carlos, São Carlos 13565-905, Brazil; campanholi@ufscar.br; 3 Graduation Program on Physical Education, Catholic University of Brasília, Brasília 71966-700, Brazil; jonatop@ucb.br; 4 Department of Kinesiology and Nutrition Sciences, University of Nevada, Las Vegas, NV 89154, USA; james.navalta@unlv.edu

**Keywords:** exercise, pulse wave velocity, arterial stiffness, ambulatory blood pressure monitoring

## Abstract

**Background/Objectives**: Long COVID (LC) has been linked to fatigue, exercise intolerance, and autonomic dysfunction, but sex-stratified data on cardiovascular responses to maximal exercise—an essential component of personalized medicine—are scarce. This study aimed to examine hemodynamic, autonomic, and functional responses during and up to 24 h after a cardiopulmonary exercise test (CPET) in young adults with and without Long COVID (LC). **Methods**: In this cross-sectional study, we assessed 38 physically active adults, who were allocated into four subgroups stratified by clinical condition (LC or control) and biological sex: control–female (CON-F; *n* = 10), LC–female (LC-F; *n* = 10), control–male (CON-M; *n* = 10), and LC–male (LC-M; *n* = 8). Outcomes included systolic (SBP) and diastolic blood pressure (DBP), heart rate (HR), cardiac output (CO), total (TPR) and peripheral vascular resistance (PVR), pulse wave velocity (PWV), augmentation index (AIx@75), and heart rate variability (HF, LF, LF/HF), assessed at rest, peak effort, recovery (1, 3, 5, 10, 30, and 60 min), and through 24 h ambulatory blood pressure monitoring (ABPM) after CPET. **Results**: SBP increase appropriately during exercise, with higher peaks in males (*p* < 0.01), and returned to baseline within 5 min across all groups. HR recovery was preserved; however, LC-F showed lower values than CON-F at 3, 5, and 10 min (126 vs. 144 bpm, *p* = 0.020; 119 vs. 136 bpm, *p* = 0.020; 94 vs. 109 bpm, *p* = 0.011), though all groups normalized by 60 min. PWV, AIx@75, TPR and PVR exhibited expected sex-related patterns without LC-related impairments. HRV indices showed transient post-exercise shifts (HF↓, LF↑, LF/HF↑). Ambulatory monitoring confirmed preserved circadian modulation, with normal systolic dipping (11–13%) and no abnormal nocturnal patterns. **Conclusions**: Young physically active adults with LC showed preserved hemodynamic, autonomic, and vascular responses during and after maximal exercise. These findings contribute to personalized medicine by showing that individualized, sex-stratified cardiovascular assessments reveal no clinically relevant impairments in this population, supporting tailored clinical decision making and exercise prescription.

## 1. Introduction

Long COVID (LC) has emerged as a major public health challenge in the post-pandemic period, characterized by persistent and potentially disabling symptoms lasting more than 12 weeks after SARS-CoV-2 infection, without an alternative explanation [1]. Common manifestations include fatigue, dyspnea, palpitations, cognitive disturbances, and reduced exercise tolerance [1,2,3]. Studies report that up to one-third of previously infected individuals may experience symptoms for as long as 24 months [4], with a substantial impact on quality of life and an increased burden of cardiovascular complaints [5,6].

Although initially associated with severe cases, LC also affects non-hospitalized and vaccinated individuals, suggesting that persistent systemic dysfunction is not exclusively dependent on the initial severity of the disease [6,7,8,9]. Recent registry data further show that the clinical presentation of patients with persistent post-COVID symptoms differs according to hospitalization status, vaccination status, and pandemic wave, with vaccinated individuals presenting fewer symptoms and a milder post-acute course compared with unvaccinated or previously hospitalized patients [10]. Within this context, the cardiovascular–autonomic axis has been a recurrent target of alterations, including sustained blood pressure (BP) elevation, increased vascular stiffness and resistance, impaired autonomic recovery, and reduced heart rate variability (HRV) [11,12,13,14].

Moreover, sex-related differences should be considered: while males are at higher risk of severe outcomes in the acute phase, females display up to a threefold higher prevalence of LC and more persistent symptom burdens [15,16]. Studies have shown that COVID-19 is associated with accelerated vascular aging, particularly in females: pulse wave velocity (PWV) increases post-COVID are more evident in females, even after adjusting for confounders [17]. Females also tend to have different autonomic modulation—both at rest and in response to stress—including higher parasympathetic indices, lower sympathetic tone at rest, and greater modulation during recovery from maximal or supramaximal exercise [18,19]. Differences in vascular compliance have also been reported, for example with less vasoconstriction and lower vascular resistance in females versus males during and after exercise [20]. Taken together, these sex-specific patterns highlight the need for stratified physiological evaluation as a cornerstone of personalized medicine, enabling more accurate characterization of individualized risk and functional capacity.

Despite the recent advances, most studies have assessed parameters at rest, limiting the detection of subtle dysfunctions that may appear only under physiological stress. Evidence suggests that LC may impair arterial compliance, peripheral resistance, and autonomic regulation during exertion, but investigations into long lasting cardiovascular and autonomic recovery following maximal exercise are lacking—particularly with follow-up as long as 24 h, and sex-based stratification [21,22]. Dynamic, individualized monitoring during and after exercise aligns with precision cardiovascular assessment, offering clinically relevant insights for personalized follow-up and tailored exercise prescription in LC.

Cardiopulmonary exercise testing (CPET) is the gold standard for assessing integrated cardiovascular, ventilatory, and autonomic responses under controlled conditions, and enables the detection of abnormalities that are not discernible at rest [23,24,25]. In individuals with LC, CPET studies have demonstrated exaggerated pressor responses, reduced peak oxygen consumption (VO_2_ peak), delayed heart rate recovery, and abnormal HRV patterns [25,26,27].

However, no studies to date have continuously evaluated hemodynamic and autonomic responses for up to 24 h after CPET in physically active young adults with LC. To address this gap, the present study aimed to characterize cardiovascular and autonomic recovery over the 24 h following maximal exercise in physically active young adults with and without LC, stratified by sex. We hypothesized that individuals with LC—particularly females—would exhibit impaired recovery, characterized by an exaggerated pressor response, delayed heart rate recovery, increased arterial stiffness and peripheral resistance, sympathetic predominance during recovery, and reduced VO_2_ peak.

## 2. Materials and Methods

### 2.1. Study Design and Ethical Approval

This cross-sectional, stratified observational study was conducted in accordance with the Declaration of Helsinki and was approved by the Human Research Ethics Committee (CAAE: 68303823.5.0000.5504). All participants provided written informed consent prior to enrollment. The study design followed recommendations for experimental research involving cardiovascular physiology in clinical populations [24,28].

### 2.2. Participants and Group Allocation

**Figure 1:**
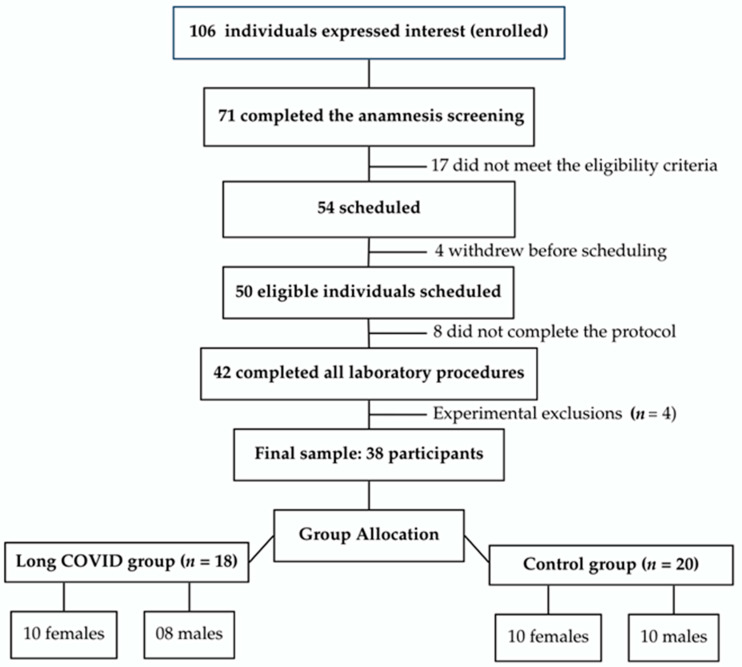
Flowchart of participant screening, eligibility, exclusions, and allocation into study subgroups. Flowchart describing participant identification (*n* = 106), screening and eligibility assessment (*n* = 54), completion of the experimental protocol (*n* = 42), reasons for exclusion (*n* = 4), and final allocation into the four study subgroups: control–female (CON-F; *n* = 10), control–male (CON-M; *n* = 10), Long COVID–female (LC-F; *n* = 10), and Long COVID–male (LC-M; *n* = 8).

A total of 106 individuals registered for the study (identification). After anamnesis screening, 54 met the eligibility criteria and were invited for laboratory assessments (screening). Of these, 42 completed the full experimental protocol, and 4 were subsequently excluded due to technical criteria (1 atypical pressor response, 2 with body fat percentage below reference thresholds, and 1 with inconsistent data). The final sample comprised 38 physically active adults aged 20–40 years. Participants were allocated into four subgroups based on sex and LC status: control–female (CON-F; *n* = 10), control–male (CON-M; *n* = 10), Long COVID–female (LC-F; *n* = 10), and Long COVID–male (LC-M; *n* = 8) (Figure 1).

Prior SARS-CoV-2 infection was confirmed through documented positive RT-PCR or antigen test results issued by accredited clinical laboratories, which all participants were required to provide during the screening process. Severity of the acute infection was determined using a structured anamnesis questionnaire that assessed clinical presentation during the acute phase, including fever, dyspnea, chest discomfort, need for medical evaluation, and temporary functional limitation. No participant required hospitalization, emergency care, oxygen therapy, or ventilatory support, indicating exclusively mild to moderate acute COVID-19 cases. For individuals allocated to the Long COVID group, all evaluations were performed ≥12 weeks after confirmed infection, in accordance with established clinical definitions of Long COVID [3]. Furthermore, inclusion criteria for the LC group were a confirmed history of SARS-CoV-2 infection, absence of hospitalization, and a physical activity level ≥ 600 MET-min/week assessed by the International Physical Activity Questionnaire (IPAQ). Exclusion criteria included pregnancy, smoking, use of β-blockers, and history of cardiovascular, pulmonary, or endocrine disease.

### 2.3. Symptom Profiling Long COVID

Participants with LC completed a standardized questionnaire detailing symptom type, duration, and intensity, based on criteria proposed by Nalbandian et al. (2021) [3]. Common symptoms assessed included fatigue, chest pain, palpitations, dyspnea, cognitive dysfunction, and effort intolerance. Symptoms were categorized by duration and frequency.

### 2.4. Menstrual and Reproductive Status of Female Participants

Based on the collected information, the individual duration of each menstrual cycle was estimated, and the predicted start date of the next cycle was calculated. Cycle phases were categorized relatively, following typical physiological patterns as described by McNulty et al. (2020) [29], with proportional adjustments for cycles shorter or longer than 28 days.

The phases were defined as follows: follicular phase (from menstruation to the estimated ovulation), ovulatory phase (around mid-cycle), and luteal phase (post-ovulation to the beginning of the next cycle). Physiological data collection was standardized to occur preferentially between the late follicular and mid-luteal phases, which are characterized by lower hormonal fluctuations and greater cardiovascular and autonomic stability [29].

### 2.5. Experimental Design

**Figure 2:**
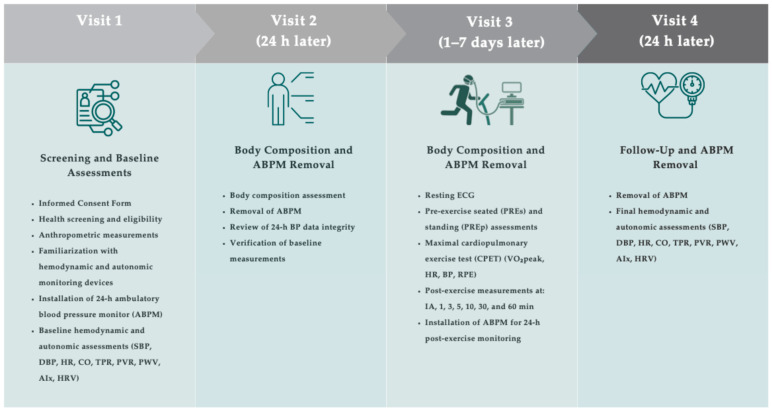
Timeline and Overview of Study Visits and Measurement Procedures. ABPM: ambulatory blood pressure monitoring; SBP: systolic blood pressure; DBP: diastolic blood pressure; HR: heart rate; CO: cardiac output; TPR: total peripheral resistance; PVR: peripheral vascular resistance; PWV: pulse wave velocity; AIx: augmentation index; HRV: heart rate variability; PREs: pre-exercise seated; PREp: pre-exercise standing; CPET: cardiopulmonary exercise testing; VO_2_ peak: peak oxygen uptake; RPE: rating of perceived exertion; IA: immediately after exercise.

The study was conducted in four sequential phases, designed to ensure methodological rigor, participant safety, and experimental consistency (Figure 2). In the first phase, recruitment and initial screening were carried out through institutional outreach and direct invitations. Participants completed a pre-screening electronic form, a detailed health history questionnaire, and complementary instruments. These tools collected data on general health status, medication use, habitual physical activity, and comorbidities, as well as prior SARS-CoV-2 infection and persistent symptoms. Based on this information, participants were classified into two groups: with (Long COVID) and without LC (Control).

The second phase included clinical assessment, anthropometry, and body composition analysis. Anthropometric measurements—height, body mass, and circumferences (waist, hip, neck, and abdomen)—were taken in triplicate using standardized techniques [30]. Body mass index and waist-to-hip ratio were calculated. Body composition was assessed using multifrequency tetrapolar bioelectrical impedance analysis (InBody 770^®^, InBody Co., Ltd., Seoul, Republic of Korea), strictly following the manufacturer’s guidelines. To minimize measurement variability, participants were instructed to maintain adequate hydration, abstain from alcohol and caffeine for at least 24 h, refrain from moderate-to-vigorous physical activity on the day prior to testing, and to void their bladder 30 min before assessment. All measurements were conducted in the morning, after a minimum 8 h overnight fast, with participants barefoot and wearing light clothing. Physical activity levels were evaluated using the Physical Activity Readiness Questionnaire (PAR-Q) [31] and the International Physical Activity Questionnaire (IPAQ) [32].

The third phase consisted of the experimental protocol involving a CPET. Before the test, resting hemodynamic parameters—heart rate (HR), systolic blood pressure (SBP), diastolic blood pressure (DBP), cardiac output (CO), pulse wave velocity (PWV), augmentation index (AIx), total peripheral resistance (TPR), and peripheral vascular resistance (PVR)—and autonomic parameters—high-frequency power (HF), low-frequency power (LF), and the LF/HF ratio—were recorded. After five minutes of supine electrocardiogram and a 10 min seated baseline collection, participants underwent a maximal CPET. Throughout the test, heart rate, BP, and perceived exertion (Borg 6–20 scale) were continuously monitored. Post-exercise, participants remained seated, and serial measurements were taken at the following intervals: immediately post-peak (IA), 1 (A1min), 3 (A3min), 5 (A5min), 10 (A10min), 30 (A30min), and 60 (A60min) minutes. Following these recordings, a 2 h ABPM device was installed to assess subacute responses.

In the final phase, 24 h after CPET, participants returned for final assessments. The ABPM device was removed, and resting hemodynamic and autonomic measurements were repeated, following the same procedures used in the earlier sessions.

### 2.6. Cardiopulmonary Exercise Testing

Ambient conditions were controlled at a temperature of 22 °C throughout all sessions. Participants were instructed to attend the CPET session wearing light clothing and appropriate footwear. Additionally, they were advised to consume a light meal at least four hours before the test, avoid stimulants and alcoholic beverages, and refrain from moderate or vigorous physical activity within 24 h before, on the day of, and after the test to prevent confounding effects on cardiovascular and autonomic responses.

The CPET was performed on a motorized treadmill (Inbramed Super ATL^®^, Porto Alegre, Brazil), following the Bruce protocol [33], characterized by incremental increases in speed and incline every three minutes. Participants were verbally encouraged to reach voluntary exhaustion and were permitted to use the side rails only for balance. BP was measured at rest, 30 s before the end of each stage, at peak effort, and at recovery intervals: immediately after, and at 3, 5, 10, 30, and 60 min post-exercise.

Cardiorespiratory variables were continuously monitored via indirect calorimetry using a VO2000^®^ gas analyzer (Medical Graphics Corporation, Minneapolis, MN, USA), providing breath-by-breath measurements of oxygen uptake (VO_2_) and carbon dioxide production (VCO_2_) every three seconds. The system does not require daily gas or syringe calibration; however, calibration procedures were carried out within the validity window established by the manufacturer. Only VO_2_ peak (mL·kg^−1^·min^−1^) was used for subsequent analyses, as other derived indices such as ventilatory thresholds or ventilatory equivalent for CO_2_ slope did not align with the study objectives. HR was continuously recorded via telemetry (Polar^®^ Electro, Kempele, Finland), and perceived exertion was assessed using the Borg scale (6–20 points). Autonomic variables (HF, LF, LF/HF) were recorded before and after the test. Participants were instructed to avoid intense physical activity in the 24 h preceding the test.

The peak effort phase was followed by an active recovery protocol consisting of 5 min of walking at 2.4 km/h and 2.5% incline, a method validated for clinical application and reproducibility (36). A CPET was considered maximal when at least three of the following criteria were met: (1) Borg score ≥ 17; (2) ≥85% of age-predicted maximal HR [208 − (0.7 × age)] (37); (3) respiratory exchange ratio ≥ 1.10; and (4) voluntary exhaustion declared by the participant. Immediate termination criteria were also established, including dizziness, angina, confusion, or severe fatigue, and physiological endpoints such as cyanosis, systolic BP drop > 10 mmHg, excessive BP elevation (>260 mmHg), diastolic BP > 115 mmHg, or absence of progressive HR increase [34].

All assessments were supervised by three trained clinical researchers: a licensed exercise professional (Bachelor of Physical Education), a certified laboratory technician, and a senior-year health sciences student specifically trained to assist with physiological testing. CPET data were exported and analyzed using Aerograph software (version 4.3), with procedures standardized for consistency and reliability.

### 2.7. Blood Pressure Assessment and 24 h Ambulatory Monitoring

During the data collection period, all sphygmomanometers and ABPM devices were calibrated in advance according to annually scheduled maintenance protocols traceable to international standards, and were inspected weekly to verify integrity and measurement accuracy.

Clinical BP measurements were obtained at multiple time points throughout the experimental protocol: pre-intervention in seated (PREs) and upright position (PREp), peak effort (MAX), immediately after exercise interruption (IA), and during recovery at 3, 5, 10, 30, and 60 min, as well as 24 h after the CPET. All resting clinical BP measurements were performed with the participant seated, back supported, feet flat on the floor, the arm relaxed at heart level, and legs uncrossed, after at least 5 min of seated rest, in accordance with the recommendations of the Brazilian Society of Cardiology [35] and the European Society of Hypertension [36]. The cuff was positioned at the midpoint of the arm, aligned with the level of the heart apex, and appropriately sized for each participant’s arm circumference, following the manufacturer’s instructions.

Measurements at PREs, PREp and at 10, 30, 60 min, and 24 h post-CPET were obtained using a validated oscillometric device for adults (Arteris AOP^®^, Cardios, São Paulo, Brazil), certified according to the International Protocol of the European Society of Hypertension [36] and measured in accordance with Brazilian Society of Cardiology [35] criteria. Measurements at MAX, IA, 3, and 5 min post-exercise were taken using the auscultatory method, with a calibrated aneroid sphygmomanometer (Hillrom™ Welch Allyn DS44, Onondaga County, NY, USA) and a Rappaport Premium stethoscope (Ningbo Sifang Medical Instruments Co., Ltd., Ningbo, China).

At the MAX moment, the cuff remained fixed to the participant’s left arm throughout the test. Inflation and stethoscope placement were initiated when the participant reported being close to the end of the exercise protocol, a determination reinforced by objective physiological indicators—such as percentage of predicted maximum heart rate (%HRmax), maximal ventilation, respiratory quotient—and the subjective perception of effort (Borg scale). In this way, the maximal BP measurement was obtained under near-exhaustion conditions. If it was not feasible to take the measurement at this point, the last reliable reading during the exercise test was used for analysis. To minimize inter-observer bias, all auscultatory measurements were performed by the same trained assessor.

Twenty-four-hour ABPM was performed using the ABPM50^®^ device (CONTEC, Qinghuangdao, China), an oscillometric technology validated according to the Association for the Advancement of Medical Instrumentation (AAMI) protocol [37,38,39]. The monitor was programmed to measure BP every 60 min, a methodological decision supported by evidence of higher compliance and reduced cuff inflation–related artifacts [40], without compromising the accuracy of mean BP values.

For ABPM data analysis, arithmetic means of SBP and DBP were calculated for the 24-h, wake, and sleep periods. Time windows were standardized as follows: wakefulness from 13:00 to 22:00 plus 09:00 of the following day (11 readings in total), and sleep from 01:00 to 06:00 (6 readings in total), in order to reduce misclassification during wake–sleep transitions, in line with the recommendations of Sánchez et al. (2020) [41]. Nocturnal dipping was calculated as:

Dipping (%) = [(mean wake BP − mean sleep BP)/mean wake BP] × 100,

as described by Bloomfield and Park (2015) [42]. Records were deemed invalid if: (a) more than 30% of readings failed; (b) there were gaps longer than 2 h without consecutive measurements; or (c) self-reported sleep duration was under 6 h or over 12 h, following Brazilian Society of Cardiology criteria. For visual representation, a continuous BP time series was plotted from 13:00 on the day of CPET until 09:00 the next day, enabling integrated visualization of acute and subacute BP responses to exercise.

During the 24 h monitoring period, participants were instructed to maintain their usual dietary and hydration habits, avoid caffeine, alcohol, and strenuous physical activity, and remain still during cuff inflations. A standardized diary was used to log sleep, meals, and daily activities.

### 2.8. Central Hemodynamics and Arterial Stiffness

Central hemodynamics and arterial compliance were assessed using an oscillometric device (Arteris AOP^®^, Cardios Sistemas Coml. Indl. Ltd.a, São Paulo, Brazil) with automated and immediate acquisition of the following parameters: PWV, AIX@75, CO, PVR, and TVR. Measurements were obtained at PREs, A10min, A30min, A60min, and A24h after the CPET.

Prior to each measurement, participants were seated in a quiet, temperature-controlled environment, with their back supported, feet flat on the floor, and legs uncrossed, for a minimum of five minutes at rest. The cuff, selected to cover approximately 80% of the participant’s arm circumference, was placed over the brachial artery according to the manufacturer’s recommendations to ensure accuracy.

The PWV was estimated indirectly from brachial pulse wave recordings, using proprietary algorithms that apply a mathematical transfer function to infer aortic pulse wave travel speed adjusted for the influence of peripheral arterial resistance. AIX@75 was also calculated indirectly, based on estimates of central systolic pressure and the amplitude of the reflected wave derived from the shape of the brachial pulse waveform. CO, PVR, and TVR were calculated using the device’s internal equations, based on pulse wave analysis and brachial BP recordings. Data extraction and processing were performed using the Arteris AOP^®^ proprietary software (version 6.384), ensuring consistency and traceability of the waveform-derived indices.

Measurements at MAX, IA, and at 3 and 5 min post-CPET could not be obtained, as the device consistently failed to produce valid readings due to the large and rapid BP fluctuations occurring between peak exercise and the initial recovery phase. Therefore, the first reliable post-exercise measurement was obtained at 10 min after CPET.

### 2.9. Heart Rate Variability (HRV) Analysis

HRV was recorded at the following time points of the experimental protocol: PREs, PREp, during the CPET, and at 10, 30, and 60 min, as well as 24 h after CPET (24 h post). HRV was analyzed at PRE, 10, 30, and 60 min, and 24 h post, with no analysis performed during exercise periods.

At all HR and HRV recording periods outside of exercise, participants were seated in a standardized position: arms relaxed, back supported, hips and knees flexed at approximately 90°, feet parallel and flat on the floor, in a quiet, temperature-controlled environment with dim lighting. Prior to each recording, spontaneous breathing was maintained for 10 min, in accordance with methodological recommendations [43,44].

Recordings were obtained using a Polar^®^ H10 heart rate transmitter (Polar Electro Oy, Kempele, Finland), validated for RR interval analysis against electrocardiography, synchronized with the Elite HRV: Wellness & Fitness^®^ application (version 5.5.9 #63, Elite HRV Inc., Asheville, NC, USA). The device recorded RR intervals at a sampling frequency of 1000 Hz. HR was processed directly in the application, whereas HRV data were exported as raw RR interval files for subsequent analysis.

Artifact detection and correction were performed manually in spreadsheet software (Excel^®^, version, 16.103.4, Microsoft Corp., Redmond, WA, USA). Only segments containing a minimum of 256 RR intervals and ≥95% normal sinus beats were retained, selected from the most stable portions of the tachogram, following established methodological recommendations [43,44]. From the PREs, 10, 30, and 60 min, and 24 h post time points, five-minute artifact-free segments containing ≥256 RR intervals were extracted for spectral analysis.

HRV analysis was performed using Kubios HRV^®^ software (version 3.4.3, Biosignal Analysis and Medical Imaging Group, University of Eastern Finland, Kuopio, Finland). Frequency-domain analysis was conducted using the Fast Fourier Transform, a non-parametric method applied to series interpolated to equidistant intervals. The following spectral components were derived: LF (0.04–0.15 Hz), HF (0.15–0.40 Hz) and the LF/HF ratio. All spectral components were expressed in normalized units (nu) to allow relative comparisons across experimental conditions.

## 3. Statistical Analysis

All statistical analyses were performed using R (RStudio 2025.05.1+513, R Core Team, Vienna, Austria), a free and open-source software environment, with the aid of the packages readxl, tidyverse, ggpubr, rstatix, dplyr, janitor, openxlsx, car, and effsize. Some statistical analyses, such as two-way and three-way ANOVA, followed by post hoc multiple comparison tests, were performed using GraphPad Prism v.8.0.1 software to confirm the results. A significance level of 5% (*p* < 0.05) was adopted for all tests. Scripts were documented and reproducible.

### 3.1. Power Sample Calculation

A sample size calculation was performed to ensure adequate statistical power to detect mean differences in systolic blood pressure (SBP) among four independent groups (CON-M, CL-M, CON-F, and CL-F). A repeated-measures analysis of variance (ANOVA) was selected, with a two-tailed significance level (α) of 0.05 and statistical power (1 − β) of 0.80. The estimation was based on previous data involving young adults (20–40 years) submitted to automated SBP measurements at baseline and at 10, 30, and 60 min after exercise, complemented by 24 h ambulatory blood pressure monitoring (ABPM) (28,29). An expected mean difference of 7 mmHg between groups and a standard deviation of 10 mmHg were assumed, with an intra-subject correlation of 0.5 and 30 repeated measurements per participant, reflecting the physiological variability typically observed in studies involving individuals with and without Long COVID.

The power analysis was conducted using G*Power v3.1.9.6 [45], selecting the F-tests family, “ANOVA: Repeated measures, within–between interaction” model, and an a priori approach, with an effect size f of 0.70. The results indicated a minimum requirement of eight participants per group (32 in total). Considering an anticipated dropout rate of 20%, the final sample target was set at ten participants per group, yielding a total recommended sample of 40 individuals.

### 3.2. Data Management and Descriptive Statistics

Raw data were imported from Excel^®^ spreadsheets and underwent a cleaning process, including variable name standardization, removal of missing or inconsistent entries, conversion of incorrect numeric formats. Continuous variables—including SBP, DBP, HR, CO, HRV parameters, PVR, TVR, PWV, AIX@75, blood glucose, body composition measures, and anthropometric measurements—were expressed as mean ± standard deviation (SD) and 95% confidence intervals (95% CI). Categorical variables (e.g., contraceptive profile, physical activity level, presence of persistent symptoms) were expressed as absolute frequency (*n*) and relative frequency (%).

### 3.3. Assumption Testing

Normality of each group was assessed using the Shapiro–Wilk test. When both groups met the assumption of normality, homogeneity of variances was tested with Levene’s test. If variances were homogeneous, independent-samples *t*-tests were applied; if heterogeneity of variances was detected, Welch’s *t*-test was used instead. When at least one group violated the assumption of normality, group comparisons were performed with the non-parametric Mann–Whitney U test. Global effects were first tested using a three-way repeated-measures ANOVA. Sphericity was assessed with Mauchly’s test; when violated (*p* < 0.05), Greenhouse–Geisser or Huynh–Feldt corrections were applied. In cases where the assumption of normality was not met for repeated measures, non-parametric alternatives were used. Specifically, the Friedman test was applied for within-subject factors, and the Aligned Rank Transform procedure (ARTool package in R, version 0.11.2) was used to allow factorial analyses with non-parametric data. Linear mixed-effects models were additionally considered when appropriate.

### 3.4. Between-Group and Between-Sex Comparisons

Comparisons between clinical status (CON vs. CL) and sex (female vs. male) were stratified into four primary contrasts: CON-F vs. CL-F, CON-M vs. CL-M, CON-F vs. CON-M, and CL-F vs. CL-M. Effect sizes for mean differences were calculated using Cohen’s d, interpreted according to conventional thresholds (small ≥ 0.2, medium ≥ 0.5, large ≥ 0.8). For dependent samples, effect sizes were corrected for repeated measures using, e.g., Cohen’s d_z_. When assumptions of equal variances were not met, Welch’s *t*-test was applied. For non-parametric tests (e.g., Mann–Whitney U), effect sizes were derived from the standardized test statistic (r = Z/√N).

### 3.5. Categorical Variable Analysis

For categorical variables, Pearson’s chi-square test was applied when expected cell counts allowed; otherwise, Fisher’s exact test was used.

### 3.6. Longitudinal Analyses

Dynamic responses over multiple time points (e.g., SBP, DBP, HR, CO, HRV, AIX@75, PWV) were analyzed using repeated-measures ANOVA, adjusted for “group” and “sex” factors. Where applicable, two-way ANOVA (clinical status × sex) or three-way ANOVA (clinical status × sex × time) models were employed. Upon identification of significant interactions or main effects, pairwise post hoc comparisons were conducted with Tukey correction to control the familywise error rate. For outcomes violating normality or homogeneity assumptions, Aligned Rank Transform (ART) procedure (ARTool package in R) was used to allow factorial analyses with non-parametric data. Furthermore, the temporal delta analyses (Δ pre–max, Δ max–immediate, Δ immediate–3 min, Δ 3–5 min, Δ 5–10 min, Δ 10–30 min, Δ 30–60 min, Δ 60 min–24 h) was used to evaluate the behavior between the temporal moments of each variable, between the groups. To compare continuous variables between groups, data distribution was first assessed for normality (Shapiro–Wilk test) and homogeneity of variances (Levene’s test). When both assumptions were satisfied (*p* > 0.05 for normality and homoscedasticity), comparisons were conducted using the Student’s *t* test for independent samples. In cases where normality was preserved but variances were unequal, the Welch’s *t* test was applied. For variables violating the normality assumption, the Mann–Whitney U test was used as a non-parametric alternative. In addition to hypothesis testing, the effect size was estimated through Cohen’s *d*, with interpretation according to conventional thresholds: negligible (<0.2), small (0.2–0.49), medium (0.5–0.79), and large (≥0.8).

### 3.7. HRV-Specific Analysis

HRV analysis was based on five-minute artifact-free segments extracted from tachograms at PRE, 10, 30, and 60 min, and 24 h post. Frequency-domain analysis used the Fast Fourier Transform to calculate low frequency (LF; 0.04–0.15 Hz), high frequency (HF; 0.15–0.40 Hz), and the LF/HF ratio, expressed in normalized units (nu). Pre-processing steps and interpolation parameters (type, order, resampling rate: X) followed the standard recommendations [43,44].

### 3.8. ABPM-Specific Analysis

ABPM data were summarized as mean SBP and DBP for 24 h, wake, and sleep periods defined by fixed time windows excluding transitions. The nocturnal dipping (%) was calculated as [(mean wake BP − mean sleep BP)/mean wake BP] × 100. Group comparisons used independent-sample *t*-tests or Mann–Whitney tests, as appropriate, with effect sizes (Cohen’s *d*). Invalid records were excluded according to predefined criteria.

## 4. Results

### 4.1. Anthropometric and Body Composition Characteristics

Female participants from both the CL group (CL-F) and the CON group (CON-F) exhibited significantly lower values for height (CL-F: 160.9 ± 6.2 cm; CL-M: 180.6 ± 7.9 cm; *p* = 0.00002), body mass (CL-F: 64.7 ± 6.3 kg; CL-M: 88.3 ± 12.0 kg; *p* = 0.00006), and neck circumference (CL-F: 33.1 ± 2.3 cm; CL-M: 38.1 ± 1.8 cm; *p* = 0.0001) when compared with male participants from both groups. There were no statistically significant differences between CL and CON groups within each sex for these variables (*p* > 0.05), as shown in Table 1.

There were no differences for body proportion indices, including waist-to-hip ratio (CL-F: 0.81 ± 0.1; CL-M: 0.90 ± 0.1; *p* = 0.055) and waist-to-height ratio (CL-F: 0.50 ± 0.04; CL-M: 0.50 ± 0.1; *p* = 0.328), between groups (*p* > 0.05) (Table 1)

### 4.2. Contraceptive Profile and Offspring in Female Participants

In the CON group (CON-F), 20% of participants (*n* = 2) did not use hormonal contraceptive methods; one reported the use of a Kyleena intrauterine device, while the other did not declare a specific method. The remaining 60% (*n* = 6) reported hormonal contraception, including one participant using a subdermal implant (*n* = 1) and five using oral contraceptives exclusively (*n* = 5). In the CL group (CL-F), the proportion of non-users of hormonal contraceptives was similar (30%, *n* = 3), whereas 70% (*n* = 7) reported exclusive use of combined oral contraceptives. Additionally, one participant in the CL-F group reported having one child (Table 2).

### 4.3. Severity of Acute COVID-19

Analysis of acute COVID-19 severity distribution between groups revealed that females in the CL group (CL-F) predominantly reported moderate symptoms (80%), whereas females in the CON group (CON-F) presented a higher frequency of mild cases (60%). Among males, both CL-M and CON-M participants reported a predominance of mild symptoms (75% and 70%, respectively). Within-group distribution revealed that 80% of females in the CL group (CL-F) experienced moderate symptoms, while in males (CL-M), mild cases predominated (75%). In the CON group, the distribution was more balanced, with 60% mild and 40% moderate among females, and 70% mild and 30% moderate among males. There were no statistically significant differences between groups (*p* = 0.169 for females; *p* = 1.000 for males) (Table 3).

### 4.4. Number and Type of Symptoms During Acute COVID-19

The most frequent reported symptoms during the acute phase were fatigue, fever, dyspnea, and cough (Table 3). Female participants in the CL group (CL-F) reported a significantly higher number of symptoms (5.1 ± 0.31) compared with CON-F (3.27 ± 0.43; *p* = 0.003). Within this group, nasal congestion (CL-F: 100% vs. CON-F: 70%) and fever (CL-F: 100% vs. CON-F: 50%) were particularly prevalent. Similarly, male participants in the CL group (CL-M) presented a higher number of symptoms (4.5 ± 0.8) compared with CON-M (2.5 ± 0.5), with statistical significance (*p* = 0.042) (Table 3).

### 4.5. History of COVID-19: Time and Frequency

The interval between the last COVID-19 diagnosis and the CPET was significantly shorter in females from the CL group (CL-F: 14.8 ± 4.11 months; 444 ± 123.2 days) compared with CON-F (27 ± 4.11 months; 855 ± 90.7 days; *p* = 0.049). There was no significant difference among males (CL-M vs. CON-M; *p* = 0.778) (Table 3). Regarding frequency, most participants reported only one confirmed positive diagnosis (laboratory or rapid test), with similar proportions across groups (CL-F: 70%; CL-M: 63%; CON-F: 70%; CON-M: 80%) (Table 3).

### 4.6. Persistent Clinical Manifestations in the CL Group

Persistent clinical manifestations reported by participants in the CL group showed distinct patterns between sexes. The mean number of persistent symptoms did not differ substantially between females (CL-F: 1.3 ± 0.2) and males (CL-M: 1.4 ± 0.2) (Table 3). In females (CL-F), the most prevalent symptoms were fatigue (20%) and hair loss (20%). In males (CL-M), memory loss (37%) and fatigue (25%) were the most frequently reported persistent manifestations (Table 3).

### 4.7. Vaccination Distribution Among Groups

In females from the CL group (CL-F), vaccine coverage was highest for Pfizer, with 40% receiving two doses and 30% three doses. Coronavac was administered to 30% (two doses), while AstraZeneca reached 20% coverage (two doses). In the CON group (CON-F), 40% of participants received two doses of Pfizer, 40% two doses of Coronavac, 20% two doses of AstraZeneca, and 20% one dose of Janssen (Table 3).

Among males in the CL group (CL-M), 37% received two doses of Pfizer and 12% three doses. Coronavac accounted for 12% (two doses), AstraZeneca for 25% (two doses), and Janssen for 37%. In the CON group (CON-M), 30% received four doses of Pfizer, 20% two doses of Coronavac, 20% two doses of AstraZeneca, and 10% one dose of Janssen. Sinovac vaccine coverage was low in both groups, with administration to 12.5% of CL-M and 10% of CON-M participants (Table 3).

### 4.8. CPET Performance and Physical Activity Level

In the analysis of completed CPET stages, no statistically significant differences were found between groups when stratified by sex (females: *p* = 0.628; males: *p* = 0.400) (Table 4). Among females, 40% of CL-F participants completed stage 3 and 50% reached stage 4, a distribution comparable to CON-F (20% and 60%, respectively). Among males, 62.5% of CL-M participants completed stage 4, compared with 50% of CON-M. Intraclass analysis showed similar distributions by sex: in CL, 50% of females and 62.5% of males completed 4 stages (*p* = 0.664), whereas in CON, the proportions were 60% and 50%, respectively (*p* = 1.000) (Table 4).

Test duration was similar between female groups (CL-F: 687.1 ± 99.2 s vs. CON-F: 686.9 ± 128.0 s; *p* = 0.996) and between male groups (CL-M: 716.8 ± 131.6 s vs. CON-M: 810.1 ± 133.4 s; *p* = 0.158). However, within the CON group, males demonstrated longer exercise duration than females (*p* = 0.049) (Table 4).

Cardiovascular responses also showed no relevant differences. The predicted maximum heart rate did not differ significantly between CL-F (188 ± 5 bpm) and CON-F (192 ± 2 bpm; *p* = 0.084), nor between males CL-M (188 ± 5 bpm) and CON-M (188 ± 4 bpm; *p* = 0.821). The 90% HR prediction showed a comparable pattern (CL-F: 169 ± 4 bpm vs. CON-F: 172 ± 2 bpm; *p* = 0.085; CL-M: 169 ± 4 bpm vs. CON-M: 170 ± 4 bpm; *p* = 0.892), with no intragroup differences (CL: *p* = 1.000; CON: *p* = 0.109). The peak HR achieved was similar between CL-F (182 ± 9 bpm; 95% CI: 175–188) and CON-F (188 ± 7 bpm; 95% CI: 184–193; *p* = 0.057), and between CL-M (187 ± 5 bpm; 95% CI: 184–191) and CON-M (184 ± 10 bpm; 95% CI: 178–191; *p* = 0.755), with no sex-based differences (Table 4). The percentage of predicted HR reached was uniformly high across subgroups (CL-F: 96.7 ± 4.2%; CL-M: 99.8 ± 2.6%; CON groups: 98–98.4%), without statistical significance (Table 4).

Resting rate pressure product (RPP) was similar across groups (CL-F: 6663 ± 1277 vs. CON-F: 7985 ± 648; *p* = 0.835; CL-M: 7072 ± 1671 vs. CON-M: 7134 ± 1183; *p* = 0.928) and between sexes within each group (CL: *p* = 0.564; CON: *p* = 0.061). At peak exercise, however, sex differences emerged: males exhibited higher peak RPP compared with females in both CL (30,310 ± 3354 vs. 25,177 ± 4866; *p* = 0.022) and CON groups (31,760 ± 3447 vs. 27,056 ± 3687; *p* = 0.008) (Table 4).

Regarding maximal effort criteria, most participants met test termination parameters (CL-F: 100%; CL-M: 87%; CON-F: 60%; CON-M: 100%). Test interruption was voluntary in all subgroups (100%). On the BORG scale, >17 scores were reported in 60% of CL-F and 62% of CL-M, compared with 40% of CON-F and 70% of CON-M individuals (Table 4). All participants achieved >90% of predicted HR, except one CON-M participant (90%). Peak RER values were similar across groups (1.1 ± 0.1) (Table 4).

Peak oxygen uptake (VO_2_ peak, mL/kg/min) was similar between CL-F (36.4 ± 7.1) and CON-F (37.7 ± 7.5; *p* = 0.696) and between CL-M (42.5 ± 9.3) and CON-M (45.9 ± 5.6; *p* = 0.328). Within groups, CON females showed significantly lower VO_2_ peak compared with CON males (*p* = 0.013). According to VO_2_ peak classification, CL-F participants were distributed as 50% “good,” 20% “regular,” and 20% “poor,” whereas CL-M were 50% “regular,” 25% “good,” and 25% “excellent” (Table 4). In the CON group, 50% of females and 60% of males were classified as “good,” with 30% and 20% reaching “regular,”, respectively; additionally, 20% of females were categorized as “poor,” while 20% of males achieved an “excellent” classification. Within-group comparisons revealed that CL-M presented a distinct pattern with higher representation in the “regular” category (50% vs. 20% in females) and presence of the “excellent” category (25%), absent in CL-F. In contrast, CON presented a more balanced sex distribution, with “excellent” observed only among males (20%) (Table 4).

Physical activity level (IPAQ) indicated that 90% of CL-F, 100% of CL-M, 90% of CON-F, and 100% of CON-M were classified as “active.” Additionally, 10% of females in both CL and CON groups were categorized as “irregularly active B” (Table 4).

### 4.9. Blood Pressure Dynamics During CPET and Recovery up to 1 h

**Figure 3:**
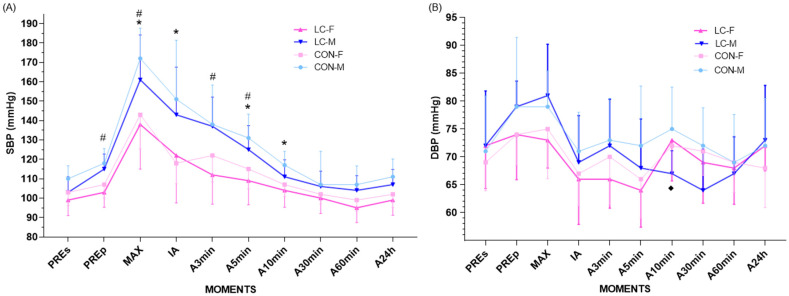
Systolic (**A**) and diastolic (**B**) blood pressure responses before, during, and after maximal exercise. Values represent mean ± standard deviation. Groups are displayed as follows: Long COVID–female (LC-F), Long COVID–male (LC-M), control–female (CON-F), and control–male (CON-M). Significant differences (three-way ANOVA, *p* < 0.05): # within-group sex differences in the Long COVID group (LC-M vs. LC-F); * within-group sex differences in the control group (CON-M vs. CON-F); ◆ between-group difference among males (LC-M vs. CON-M).

The analysis of SBP during CPET showed consistent hemodynamic patterns, with significant differences between sexes but not between CL and CON groups. At rest (seated), no significant differences were observed between CL-F (99.4 ± 7.8 mmHg; 95% CI: 92.2–106.6) and CON-F (103.5 ± 7.2 mmHg; 95% CI: 96.8–110.2; *p* = 0.192), nor between CL-M (102.6 ± 7.4 mmHg; 95% CI: 95.5–109.7) and CON-M (109.9 ± 6.5 mmHg; 95% CI: 103.3–116.5; *p* = 0.391) (Figure 3A). However, in the pre-standing phase, CL females presented significantly lower SBP compared with CL males (103.1 ± 7.0 mmHg; 95% CI: 96.5–109.7 vs. 114.8 ± 8.8 mmHg; 95% CI: 106.6–123.0; *p* = 0.041) (Figure 3A).

At maximal exertion, males reached higher SBP values in both groups (CON-M: 171.7 ± 16.5 mmHg; 95% CI: 156.7–186.7 vs. CON-F: 143.1 ± 20.8 mmHg; 95% CI: 123.9–162.3; *p* = 0.007; CL-M: 161.3 ± 16.8 mmHg; 95% CI: 145.8–176.8 vs. CL-F: 136.9 ± 22.6 mmHg; 95% CI: 116.2–157.6; *p* = 0.005) (Figure 3A).

During recovery, SBP decreased similarly across groups, with a significant time effect (F = 32.95, *p* < 0.001) and no group × time interaction (F = 0.53, *p* = 0.853). All subgroups returned to pre-exercise values within 5 min (*p* > 0.05). Recovery was faster in females (immediately post-exercise in CL-F; 3 min in CON-F) compared to males (≤5 min). Immediately after exercise, CON-F exhibited lower SBP than CON-M (119.8 ± 11.9 mmHg; 95% CI: 108.8–130.8 vs. 145.1 ± 28.3 mmHg; 95% CI: 119.6–170.6; *p* = 0.019) (Figure 3A). At 3 min, CL-F presented lower SBP compared with CL-M (112.2 ± 15.6 mmHg vs. 132.8 ± 12.8 mmHg; *p* = 0.032). At 5 min, sex differences persisted in both groups (CL-F: 109.4 ± 13.4 mmHg; 95% CI: 96.8–122.0 vs. CL-M: 124.8 ± 11.0 mmHg; 95% CI: 114.5–135.1; *p* = 0.047; CON-F: 115.2 ± 9.7 mmHg; 95% CI: 106.0–124.4 vs. CON-M: 130.5 ± 12.8 mmHg; 95% CI: 118.8–142.2; *p* = 0.033) (Figure 3A). At 10 min, differences remained only in the CON group (CON-F: 106.7 ± 6.8 mmHg; 95% CI: 100.3–113.1 vs. CON-M: 116.6 ± 6.8 mmHg; 95% CI: 110.2–123.0; *p* = 0.043) (Figure 3A). No significant differences were observed at 30 min, 60 min, or 24 h. At 60 min, SBP dropped below pre-seated values in most groups, with slightly lower means in females (CL-F: –4.5 mmHg; CON-F: –4.2 mmHg; CL-M: +0.2 mmHg; CON-M: –3.1 mmHg) (Figure 3A). Temporal SBP deltas (Δ pre–max, Δ max–immediate, Δ immediate–3 min, Δ 3–5 min, Δ 5–10 min, Δ 10–30 min, Δ 30–60 min, Δ 60 min–24 h) showed no significant differences between groups or sexes.

For DBP, hemodynamic patterns were homogeneous, with no significant differences between groups (ANOVA group × time, *p* > 0.05 for all comparisons) (Figure 3B). At baseline (seated), values were comparable across sexes and groups (CON-F: 69.2 ± 5.7 mmHg; 95% CI: 63.8–74.6; CL-F: 72.2 ± 7.2 mmHg; 95% CI: 65.5–78.9; CON-M: 71.8 ± 9.8 mmHg; 95% CI: 62.9–80.7; CL-M: 70.6 ± 7.7 mmHg; 95% CI: 63.3–77.9; *p* = 0.986) (Figure 3B). At maximal exertion, DBP remained stable across groups (CON-F: 74.6 ± 8.7 mmHg; 95% CI: 66.4–82.8; CL-F: 73.8 ± 6.2 mmHg; 95% CI: 67.9–79.7; CON-M: 80.6 ± 9.9 mmHg; 95% CI: 71.6–89.6; CL-M: 79.0 ± 7.2 mmHg; 95% CI: 72.1–85.9; *p* = 0.534) (Figure 3B). During recovery, values remained comparable, except at 10 min, when CON-M showed lower DBP compared with CL-M (66.9 ± 4.1 mmHg; 95% CI: 63.1–70.7 vs. 74.6 ± 5.8 mmHg; 95% CI: 69.1–80.1; *p* = 0.031) (Figure 3B). No other significant differences were observed at 30 min, 60 min, or 24 h.

Temporal DBP deltas showed no significant between-group or sex-related differences across all recovery intervals (Δ pre–max, Δ max–immediate, Δ immediate–3 min, Δ 3–5 min, Δ 5–10 min, Δ 10–30 min, Δ 30–60 min, Δ 60 min–24 h), confirming comparable cardiovascular recovery patterns in all conditions.

### 4.10. Heart Rate (HR) Responses

HR responses demonstrated distinct patterns between groups, with sex-related differences observed at specific time points. Three-way ANOVA revealed a significant effect of time (*p* < 0.0001) and group condition (CON vs. CL, *p* < 0.0001), as well as a significant interaction between sex and clinical condition (male vs. female × CON vs. CL, *p* < 0.0001). No significant interactions were found for time × sex (*p* = 0.2425), time × group condition (*p* = 0.685), or time × sex × condition (*p* = 0.982), indicating similar temporal HR patterns across groups and sexes.

**Figure 4:**
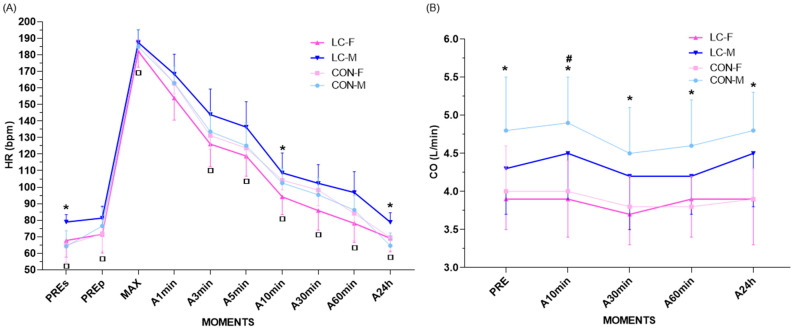
Heart rate (**A**) and cardiac output (**B**) responses before, during, and after maximal exercise. Values represent mean ± standard deviation. Groups include Long COVID–female (LC-F), Long COVID–male (LC-M), control–female (CON-F), and control–male (CON-M). Significant differences (three-way ANOVA, *p* < 0.05): # within-group sex differences in the Long COVID group (LC-M vs. LC-F); * within-group sex differences in the control group (CON-M vs. CON-F); ◻ between-group difference in females (LC-F vs. CON-F).

At pre-test rest (seated), CON-F participants exhibited significantly higher HR (78.9 ± 4.4 bpm; 95% CI: 75.9–81.9) compared with CON-M (64.2 ± 9.4 bpm; *p* = 0.003) and CL-F (67.7 ± 10.0 bpm; *p* = 0.035). Among males, no significant difference was observed between CL-M (65.0 ± 10.4 bpm; 95% CI: 56.1–73.9) and CON-M (64.2 ± 9.4 bpm; 95% CI: 56.9–71.5; *p* = 0.999) (Figure 4A).

During CPET, all groups achieved comparable maximal HR values (CL-F: 182.1 ± 9.6 bpm, 95% CI: 174.5–189.7; CL-M: 187.9 ± 5.2 bpm, 95% CI: 183.0–192.8; CON-F: 187.4 ± 7.7 bpm, 95% CI: 181.7–193.1; CON-M: 184.9 ± 10.4 bpm, 95% CI: 176.5–193.3) (Figure 4A).

During recovery, CL-F demonstrated consistently lower HR compared with CON-F at multiple time points (Figure 4A):

- Time point at 3 min: CL-F: 126.0 bpm (95% CI: 116.4–135.6) vs. CON-F: 143.8 bpm (95% CI: 132.3–155.3); *p* = 0.020.
- Time point at 5 min: CL-F: 118.8 bpm (95% CI: 109.6–128.0) vs. CON-F: 136.3 bpm (95% CI: 125.0–147.6); *p* = 0.020.
- Time point at 10 min: CL-F: 94.1 bpm (95% CI: 86.4–101.8) vs. CON-F: 108.6 bpm (95% CI: 99.6–117.6); *p* = 0.011.
- Time point at 30 min: CL-F: 85.8 ± 11.9 bpm (95% CI: 76.3–95.3) vs. CON-F: 102.3 ± 11.3 bpm (95% CI: 93.0–111.6).
- Time point at 60 min: CL-F: 78.1 ± 11.5 bpm (95% CI: 68.6–87.6) vs. CON-F: 96.7 ± 12.6 bpm (95% CI: 87.1–106.3); *p* = 0.028.
- Time point at 24 h: CL-F: 69.1 ± 8.2 bpm (95% CI: 62.9–75.3) vs. CON-F: 78.8 ± 5.7 bpm (95% CI: 74.1–83.5); *p* = 0.028.

Male participants (CL-M and CON-M) exhibited more homogeneous HR recovery, with no significant differences between groups. However, sex-based differences were identified within groups (Figure 4A):

- CON group: CON-F had higher HR compared with CON-M at 3 min (143.8 ± 15.5 bpm vs. 133.5 ± 12.4 bpm; *p* = 0.030), 5 min (136.3 ± 15.3 bpm vs. 125.0 ± 14.1 bpm; *p* = 0.018), and 24 h (78.8 ± 5.7 bpm vs. 64.6 ± 7.6 bpm; *p* = 0.001).
- CL group: CL-M exhibited higher HR than CL-F at 10 min (104.2 ± 5.9 bpm vs. 94.1 ± 10.7 bpm; *p* = 0.044) and at 30 min (98.2 ± 9.6 bpm vs. 85.8 ± 11.9 bpm; *p* = 0.013).

All groups returned to baseline HR values by 60 min, with the exception of CON-M, who maintained elevated values compared with pre-test rest (86.1 ± 8.8 bpm; 95% CI: 79.3–92.9; *p* = 0.0084) (Figure 4A). At 24 h, all groups had normalized HR to pre-CPET levels. Delta analyses of HR recovery (Δ pre–max, Δ 1–3 min, Δ 3–5 min, Δ 5–10 min, Δ 10–30 min, Δ 30–60 min, Δ 60 min–24 h) revealed no significant differences within groups or between sexes.

### 4.11. Cardiac Output (CO)

CO demonstrated consistent patterns across groups, with significant sex-related differences but no clinically relevant divergence between CON and CL conditions when analyzed by sex (Figure 4B). Three-way ANOVA confirmed a predominant sex effect (F = 73.3; *p* < 0.0001), without a group × sex interaction *(p* = 0.06), indicating that differences between males and females persisted independently of group allocation. No significant time effects were observed for either group. Post hoc analysis indicated that in the CON group, females exhibited significantly lower CO at rest (CON-F: 4.0 ± 0.6 L/min; 95% CI: 3.4–4.6) compared with males (CON-M: 4.8 ± 0.7 L/min; 95% CI: 4.2–5.4; *p* = 0.004). In contrast, no differences were observed at rest within the CL group (CL-F: 3.9 ± 0.4 L/min; 95% CI: 3.5–4.3 vs. CL-M: 4.3 ± 0.6 L/min; 95% CI: 3.7–4.9; *p* = 0.748) (Figure 4B).

At 10 min post-exercise, both groups demonstrated significant sex differences, with females presenting lower CO values compared with males (CON-F: 4.0 ± 0.4 L/min; 95% CI: 3.6–4.4 vs. CON-M: 4.9 ± 0.6 L/min; 95% CI: 4.4–5.4; *p* = 0.001; CL-F: 3.9 ± 0.5 L/min; 95% CI: 3.4–4.4 vs. CL-M: 4.5 ± 0.6 L/min; 95% CI: 3.9–5.1; *p* = 0.030) (Figure 4B). At 30 min and 60 min, sex differences persisted only in the CON group, with females presenting lower CO (30 min: CON-F: 3.8 ± 0.4 L/min; 95% CI: 3.4–4.2 vs. CON-M: 4.5 ± 0.6 L/min; 95% CI: 4.0–5.0; *p* = 0.014; 60 min: CON-F: 3.8 ± 0.4 L/min; 95% CI: 3.4–4.2 vs. CON-M: 4.6 ± 0.6 L/min; 95% CI: 4.1–5.1; *p* = 0.005) (Figure 4B). At 24 h recovery, CON-F maintained significantly lower CO values compared with CON-M (CON-F: 3.9 ± 0.4 L/min; 95% CI: 3.5–4.3 vs. CON-M: 4.8 ± 0.5 L/min; 95% CI: 4.4–5.2; *p* = 0.022). In the CL group, no statistically significant differences were observed at this time point (CL-F: 3.9 ± 0.6 L/min vs. CL-M: 4.5 ± 0.7 L/min; *p* = 0.060) (Figure 4B).

Temporal delta analyses (Δ pre–max, Δ max–immediate, Δ immediate–3 min, Δ 3–5 min, Δ 5–10 min, Δ 10–30 min, Δ 30–60 min, Δ 60 min–24 h) revealed no significant differences between groups or sexes (all *p* > 0.05), indicating comparable hemodynamic recovery profiles across conditions.

### 4.12. Pulse Wave Velocity (PWV)

**Figure 5:**
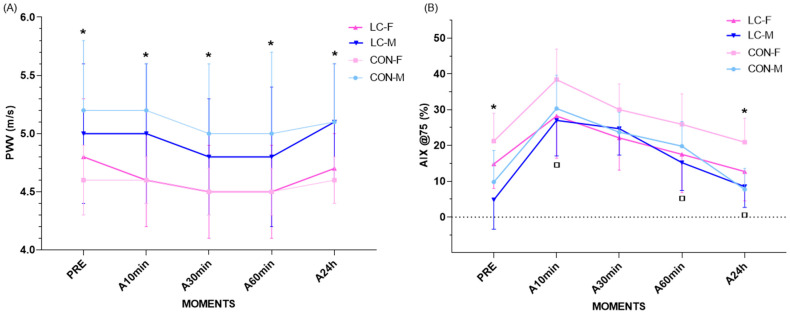
Central hemodynamic (**A**) and arterial stiffness (**B**) responses following maximal exercise. Values are presented as mean ± standard deviation. Groups include Long COVID–female (LC-F), Long COVID–male (LC-M), control–female (CON-F), and control–male (CON-M). Significant differences (three-way ANOVA, *p* < 0.05): * within-group sex differences in the control group (CON-M vs. CON-F); ◻ between-group difference in females (LC-F vs. CON-F).

PWV [90% CI] demonstrated homogeneous patterns across groups, with no significant group × time interactions (CON-F vs. CL-F: *p* = 0.858; CON-M vs. CL-M: *p* = 0.660; CON-F vs. CON-M: *p* = 0.956; CL-F vs. CL-M: *p* = 0.215), indicating that CL did not influence the temporal variation of PWV (Figure 5A). The only relevant difference was observed between sexes in the CON group, where females consistently exhibited significantly lower PWV compared with males across all analyses (*p* = 0.001). This pattern was not observed in the CL group (*p* = 0.190) (Figure 5A).

At rest, CON-F presented significantly lower brachial PWV compared with CON-M (CON-F: 4.6 ± 0.3 m/s; 95% CI: 4.3–4.9 vs. CON-M: 5.2 ± 0.6 m/s; 95% CI: 4.7–5.7; *p* = 0.010). In contrast, CL-F and CL-M presented similar values (CL-F: 4.8 ± 0.5 m/s; 95% CI: 4.3–5.3 vs. CL-M: 5.0 ± 0.6 m/s; 95% CI: 4.4–5.6; *p* = 0.960) (Figure 5A).

During recovery, the same pattern persisted in the CON group, with females maintaining significantly lower PWV compared with males at all time points:

- Time point at 10 min: CON-F: 4.6 ± 0.2 m/s (95% CI: 4.4–4.8) vs. CON-M: 5.2 ± 0.4 m/s (95% CI: 4.8–5.6); *p* = 0.010.
- Time point at 30 min: CON-F: 4.5 ± 0.2 m/s (95% CI: 4.3–4.7) vs. CON-M: 5.0 ± 0.6 m/s (95% CI: 4.5–5.5); *p* = 0.041.
- Time point at 60 min: CON-F: 4.5 ± 0.2 m/s (95% CI: 4.3–4.7) vs. CON-M: 5.0 ± 0.7 m/s (95% CI: 4.4–5.6); *p* = 0.030.
- Time point at 24 h: CON-F: 4.6 ± 0.2 m/s (95% CI: 4.4–4.8) vs. CON-M: 5.1 ± 0.5 m/s (95% CI: 4.7–5.5); *p* = 0.006.

No significant differences were observed between sexes in the CL group at any time point. Temporal delta analyses (Δ pre–max, Δ max–immediate, Δ immediate–3 min, Δ 3–5 min, Δ 5–10 min, Δ 10–30 min, Δ 30–60 min, Δ 60 min–24 h) revealed no significant differences between groups or sexes, confirming comparable recovery trajectories under all conditions.

### 4.13. Augmentation Index (AIx@75)

AIx@75 demonstrated consistent sex-related differences, with a significant main effect of time (F = 49.80; *p* < 0.001). No significant group × time interactions were observed (CON-F vs. CL-F: *p* = 0.681; CON-M vs. CL-M: *p* = 0.703; CON-F vs. CON-M: *p* = 0.368; CL-F vs. CL-M: *p* = 0.261), indicating similar temporal trajectories between CL and CON. All groups returned to baseline values within 60 min, with females showing faster recovery (30 min) (Figure 5B).

At baseline, pre-CPET AIx@75 values were significantly higher in females compared with males in both groups (CON-F: 21.2 ± 7.8% vs. CON-M: 9.8 ± 8.8%; *p* = 0.040; CL-F: 14.8 ± 6.8% vs. CL-M: 4.8 ± 8.2%; *p* = 0.003) (Figure 5B).

At 10 min post-CPET, AIx@75 increased relative to baseline across all groups, with a significant difference between CL-F and CON-F (CON-F: 38.4 ± 8.5%; 95% CI: 30.6–46.2 vs. CL-F: 28.2 ± 11.8%; 95% CI: 17.4–39.0; *p* = 0.040), a pattern not observed in males (CON-M: 30.3 ± 9.3% vs. CL-M: 27.0 ± 9.9%; *p* = 0.910) (Figure 5B). Subsequently, AIx declined gradually, returning to baseline within 30 min for females and up to 60 min for males in both groups. At 60 min, a significant difference persisted between groups among females (CON-F: 25.9 ± 8.5%; 95% CI: 18.1–33.7 vs. CL-F: 17.5 ± 10.7%; 95% CI: 7.6–27.4; *p* = 0.031) (Figure 5B).

At 24 h, significant differences were observed between sexes and groups. In the CON group, females maintained higher AIx@75 compared with males (CON-F: 20.9 ± 6.7%; 95% CI: 14.7–27.1 vs. CON-M: 7.7 ± 5.9%; 95% CI: 2.4–13.0; *p* = 0.002). Moreover, CON-F values were higher compared with CL-F (CON-F: 20.9 ± 6.7%; 95% CI: 14.7–27.1 vs. CL-F: 12.7 ± 8.2%; 95% CI: 5.3–20.1; *p* = 0.030). Conversely, within females, CL-F values were significantly lower than CON-F at this time point (*p* = 0.030) (Figure 5B).

No significant differences were found in temporal delta analyses (Δ pre–max, Δ max–immediate, Δ immediate–3 min, Δ 3–5 min, Δ 5–10 min, Δ 10–30 min, Δ 30–60 min, Δ 60 min–24 h), reinforcing that recovery patterns were comparable across groups and sexes.

### 4.14. Total Vascular Resistance (TVR)

**Figure 6:**
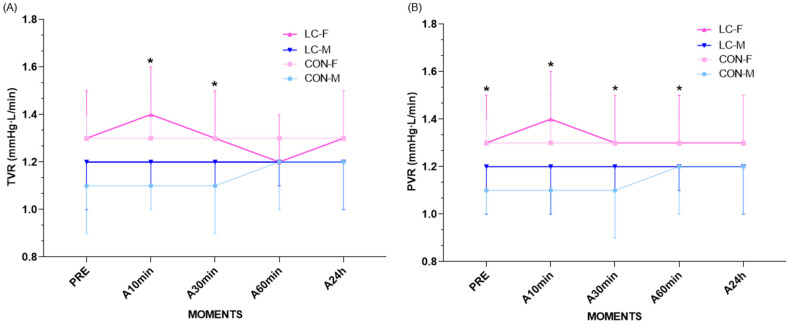
Total (**A**) and peripheral (**B**) vascular resistance responses following maximal exercise. Values are presented as mean ± standard deviation. Groups include Long COVID–female (LC-F), Long COVID–male (LC-M), control–female (CON-F), and control–male (CON-M). Significant differences (three-way ANOVA, *p* < 0.05): * within-group sex differences in the control group (CON-M vs. CON-F).

TVR exhibited homogeneous patterns across groups, with consistent sex-related variations. ANOVA confirmed a main effect of sex (F(1,170) = 40.3; *p* < 0.0001), without a group effect (F(1,170) = 1.35; *p* = 0.2469) or group × time interaction (F(4,170) = 0.269; *p* = 0.8973) (Figure 6A).

At rest, females and males in both groups demonstrated comparable TVR values with no significant sex- or group-related differences (CON-F: 1.3 ± 0.1 mmHg·min/L; 95% CI: 1.2–1.4 vs. CON-M: 1.1 ± 0.2; 95% CI: 0.9–1.3; *p* = 0.140; CL-F: 1.3 ± 0.2; 95% CI: 1.1–1.5 vs. CL-M: 1.2 ± 0.2; 95% CI: 1.0–1.4; *p* = 0.802) (Figure 6A).

At 10 min and 30 min post-CPET, CON-F participants exhibited significantly higher TVR compared with CON-M (**10 min: CON-F: 1.3 ± 0.2; 95% CI: 1.1–1.5 vs. CON-M: 1.1 ± 0.1; 95% CI: 1.0–1.2; *p* = 0.006; 30 min: CON-F: 1.3 ± 0.1; 95% CI: 1.2–1.4 vs. CON-M: 1.1 ± 0.2; 95% CI: 0.9–1.3; *p* = 0.001). No differences were observed between sexes in the CL group. At 60 min and 24 h, no significant differences were found between groups or sexes (Figure 6A).

Temporal delta analysis (Δ pre–max, Δ max–immediate, Δ immediate–3 min, Δ 3–5 min, Δ 5–10 min, Δ 10–30 min, Δ 30–60 min, Δ 60 min–24 h) revealed no significant differences by group or sex (all *p* > 0.05), reinforcing the homogeneity of patterns across recovery phases.

### 4.15. Peripheral Vascular Resistance (PVR)

PVR after CPET demonstrated consistent sex-related differences, without significant influence of clinical condition (CL). ANOVA confirmed a main effect of sex (F = 46.9; *p* < 0.0001), with no significant group × sex × time interaction (F = 0.316; *p* = 0.867) (Figure 6B).

At rest, CON-F exhibited significantly higher PVR compared with CON-M (CON-F: 1.3 ± 0.1 mmHg·min/L; 95% CI: 1.2–1.4 vs. CON-M: 1.1 ± 0.1; 95% CI: 1.0–1.2; *p* = 0.045). In contrast, no sex-based differences were found within the CL group (CL-F: 1.3 ± 0.2 vs. CL-M: 1.2 ± 0.2; *p* = 0.645) (Figure 6B).

At 10 min post-exercise, sex differences persisted only in the CON group (CON-F: 1.3 ± 0.2; 95% CI: 1.1–1.5 vs. CON-M: 1.1 ± 0.1; 95% CI: 1.0–1.2; *p* = 0.040), while no significant differences were observed in CL (CL-F: 1.4 ± 0.2; 95% CI: 1.2–1.6 vs. CL-M: 1.2 ± 0.2; 95% CI: 1.0–1.4; *p* = 0.330) (Figure 6B). At subsequent time points, females maintained higher PVR compared with males in the CON group (30 min: CON-F: 1.3 ± 0.2; 95% CI: 1.1–1.5 vs. CON-M: 1.1 ± 0.2; 95% CI: 0.9–1.3; *p* = 0.030; 60 min: CON-F: 1.3 ± 0.1; 95% CI: 1.2–1.4 vs. CON-M: 1.2 ± 0.2; 95% CI: 1.0–1.4; *p* = 0.035). At 24 h, no significant differences were found between groups or sexes (Figure 6B).

Temporal delta analyses (Δ pre–10 min, Δ 10–30 min, Δ 60–24 h) revealed no significant differences between groups; for example, Δ pre–10 min: CON-F: 0.016 ± 0.157 vs. CL-F: 0.042 ± 0.190; *p* = 0.743. These findings indicate that PVR modulation was predominantly sex-driven, without additional impact of CL status across recovery.

### 4.16. Heart Rate Variability (HRV)

#### 4.16.1. High-Frequency Component (HF)

Analysis of HRV in the frequency domain, specifically the HF component, revealed distinct sex-related patterns and homogeneity between CON and CL groups.

**Figure 7:**
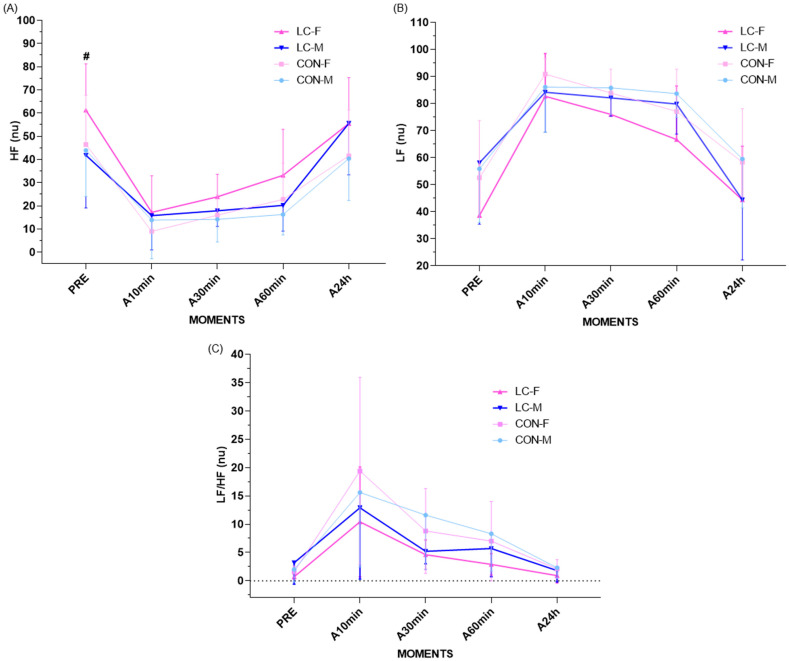
High-Frequency Component (HF) (**A**), Low-Frequency Component (LH) (**B**) and LF/HF Ratio (**C**) modulation responses following maximal exercise. Values are presented as mean ± standard deviation. Groups include Long COVID–female (LC-F), Long COVID–male (LC-M), control–female (CON-F), and control–male (CON-M). Significant differences (three-way ANOVA, *p* < 0.05): # within-group sex differences in the Long COVID group (LC-M vs. LC-F).

ANOVA indicated a significant time effect (F = 26.9; *p* < 0.0001) and sex effect (F = 10.1; *p* = 0.0017), with no group × time interaction (F = 0.315; *p* = 0.9038). Temporal differences were evident only among females, with significantly elevated HF values at 10 and 30 min post-CPET, returning to baseline by 60 min (Figure 7A).

At rest, CL-F exhibited significantly higher HF values compared with CL-M (CL-F: 61.3 ± 20.0 nu; 95% CI: 42.8–79.8 vs. CL-M: 41.8 ± 22.7 nu; 95% Cl: 20.8–62.8 nu; *p* = 0.027). In contrast, no differences were observed within the CON group (CON-F: 46.5 ± 21.2 nu; 95% CI: 26.9–66.1 vs. CON-M: 43.9 ± 19.8 nu; 95% CI: 25.6–62.2; *p* = 0.964) (Figure 7A).

At 10 min recovery, females in both groups exhibited reduced HF relative to pre-CPET (CL-F: 17.2 ± 15.8 nu; 95% CI: 2.9–31.5; CON-F: 9.0 ± 5.8 nu; 95% CI: 3.5–14.5; *p* = 0.166), with no differences between groups. Among males, HF values were comparable (CL-M: 13.9 ± 16.7 nu; 95% CI: 0.0–27.8 vs. CON-M: 15.8 ± 14.8 nu; 95% CI: 2.7–28.9; *p* = 0.962). At 30 min, HF remained elevated in CL-F (23.9 ± 9.7 nu; 95% CI: 15.0–32.8) versus CON-F (16.0 ± 8.8 nu; 95% CI: 7.9–24.1; *p* = 0.077). Males maintained comparable values across groups (CL-M: 17.9 ± 6.8 nu vs. CON-M: 14.2 ± 9.8 nu; *p* = 0.962). At 60 min, values in females returned to baseline (CL-F: 33.2 ± 19.8 nu; 95% CI: 14.0–52.4 vs. CON-F: 55.5 ± 19.8 nu; 95% CI: 37.4–73.6; *p* = 0.318). Males also had stable values without group differences (CL-M: 20.2 ± 11.1 nu vs. CON-M: 16.3 ± 8.8 nu; *p* = 0.858) (Figure 7A).

At 24 h, all groups exhibited values similar to pre-CPET with no sex or group differences (CL-M: 55.6 ± 22.2 nu vs. CON-M: 40.4 ± 18.1 nu; *p* = 0.120; CON-F: 41.6 ± 19.8 nu vs. CL-F: 55.5 ± 19.8 nu; *p* = 0.169) (Figure 7A). Temporal delta analyses revealed no significant differences between groups or sexes across all recovery intervals.

#### 4.16.2. Low-Frequency Component (LF)

The LF component, reflecting sympathetic modulation, showed physiological sex-related variability but homogeneity across groups (Figure 7B). ANOVA confirmed a main time effect (F = 27.5; *p* < 0.0001) with no group × time interaction (F = 0.254; *p* = 0.9372). Temporal changes were evident only in females, who displayed reduced LF values post-CPET (10 and 30 min) before returning to baseline at 60 min (Figure 7B).

At rest, no differences were observed among groups or sexes (CL-F: 38.6 ± 20.0 nu vs. CON-F: 52.5 ± 21.2 nu; *p* = 0.197; CL-M: 58.1 ± 22.7 nu vs. CON-M: 55.9 ± 19.8 nu; *p* = 0.978). At 10 min post-CPET, both female groups exhibited sharp increases compared with pre-exercise (CL-F: 82.7 ± 15.8 nu; 95% CI: 68.4–97.0 vs. CON-F: 90.9 ± 5.8 nu; 95% CI: 85.4–96.4; *p* = 0.166), whereas males remained similar (CON-M: 86.1 ± 16.7 nu, 95% CI: 72.2–100.0 vs. CL-M: 84.2 ± 14.8 nu, CI 95%: 71.1–97.3; *p* = 0.962). At 30 min, female values remained higher than baseline but without between-group differences (CON-F: 83.9 ± 8.8 nu; 95% CI: 75.8–92.0 vs. CL-F: 76.0 ± 9.7 nu; 95% CI: 67.1–84.9; *p* = 0.070). Males remained homogeneous (CON-M: 85.8 ± 9.8 nu, CI 95%: 76.9–947 vs. CL-M: 82.1 ± 6.8 nu, CI 95%: 75.9–88.3; *p* = 0.867). At 60 min, all groups returned to baseline (CON-F: 77.1 ± 15.6 nu vs. CL-F: 66.7 ± 19.8 nu; *p* = 0.318; CON-M: 83.7 ± 8.8 nu vs. CL-M: 79.8 ± 11.1 nu; *p* = 0.858) (Figure 7B).

At 24 h, LF values were comparable to baseline with no sex or group difference (CL-F: 44.4 ± 19.8 nu vs. CON-F: 58.3 ± 19.8 nu; *p* = 0.169; CL-M: 44.4 ± 22.2 nu vs. CON-M: 59.5 ± 18.1 nu; *p* = 0.120). Temporal deltas showed no group- or sex-related significance across recovery periods (Figure 7B).

#### 4.16.3. LF/HF Ratio

The LF/HF ratio revealed distinct physiological sex-related patterns with overall homogeneity between CON and CL. ANOVA confirmed a main effect of time (F = 16.9; *p* < 0.0001), but no group × time (F = 0.479; *p* = 0.7918) or sex × time interactions (F = 0.600; *p* = 0.700) (Figure 7C).

At rest, no significant differences were noted (CON-F: 1.6 ± 1.0; 95% CI: 0.9–2.3 vs. CON-M: 2.0 ± 2.2; 95% CI: 0.1–3.9; *p* = 0.914; CL-F: 0.7 ± 0.4; 95% CI: 0.4–1.0 vs. CL-M: 3.2 ± 3.8; 95% CI: 0.3–6.7; *p* = 0.062). ANOVA revealed a significant condition effect (F = 9.15; *p* = 0.002), though not for sex (F = 1.24; *p* = 0.266) (Figure 7C).

At 10 min post-CPET, all groups exhibited increased LF/HF ratios compared with baseline, though without statistical significance. A significant time × condition interaction (F = 2.33; *p* = 0.0435) was observed only in CON-F, without sex interaction (CL-F: 10.4 ± 9.7 vs. CON-F: 19.4 ± 16.5; *p* = 0.177). Males remained similar (CL-M: 12.9 ± 12.6 vs. CON-M: 15.6 ± 13.2; *p* = 0.907) (Figure 7C).

During later recovery, ratios declined progressively across all groups, returning to baseline within 30 min. At 24 h, all groups exhibited values similar to pre-CPET with no differences (CL-F: 0.91 ± 1.30 vs. CON-F: 2.1 ± 1.6; *p* = 0.303; CL-M: 1.8 ± 2.0 vs. CON-M: 2.3 ± 1.9; *p* = 0.959) (Figure 7C). Delta analyses revealed no significant temporal changes between groups or sexes, confirming comparable autonomic recovery patterns.

#### 4.16.4. Ambulatory Blood Pressure (ABPM) Dynamics (24 h)

**Figure 8:**
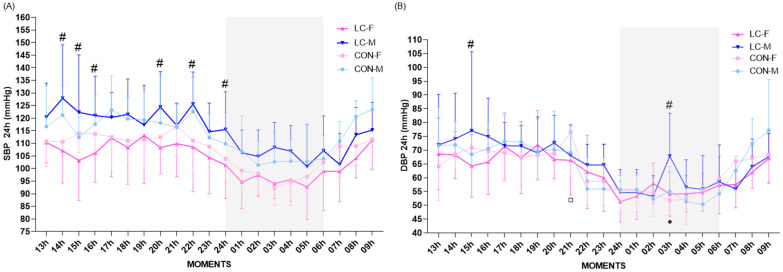
Ambulatory systolic (**A**) and diastolic (**B**) blood pressure responses over 24 h following maximal exercise. Values represent mean ± standard deviation for Long COVID–female (LC-F), Long COVID–male (LC-M), control–female (CON-F), and control–male (CON-M). The gray shaded area indicates the nocturnal (sleep) period during the 24-hour ambulatory blood pressure monitoing. Significant differences (three-way ANOVA, *p* < 0.05): # within-group sex differences in the Long COVID group (LC-M vs. LC-F); ◆ between-group differences among males (LC-M vs. CON-M); ◻ between-group differences among females (LC-F vs. CON-F).

Twenty-four-hour ABPM revealed physiological patterns, with sex-related variability and limited effects of CL. For SBP, CL-F participants exhibited values within the expected physiological range, with no significant differences compared with CON-F during wakefulness (CL-F: 108.2 ± 3.2 mmHg; 95% CI: 105.9–110.5 vs. CON-F: 111.8 ± 3.7 mmHg; 95% CI: 109.1–114.4; *p* = 0.510) or sleep (CL-F: 95.5 ± 2.1 mmHg; 95% CI: 93.3–97.8 vs. CON-F: 97.3 ± 3.4 mmHg; 95% CI: 93.7–100.9; *p* = 0.807) (Figure 8A).

Intragroup comparisons revealed that CL-F had lower SBP than CL-M, both during wakefulness (CL-M: 120.7 ± 3.7 mmHg; 95% CI: 118.1–123.4; *p* = 0.030) and sleep (CL-M: 105.7 ± 2.6 mmHg; 95% CI: 102.9–108.5; *p* = 0.045). In contrast, no significant sex differences were observed within the CON group (wakefulness: *p* = 0.411; sleep: *p* = 0.760) (Figure 8A). For 24 h mean SBP, CL-F presented significantly lower values compared with CON-F (CL-F: 104.0 ± 13.3 mmHg; 95% CI: 102.1–106.0 vs. CON-F: 107.3 ± 11.5 mmHg; 95% CI: 105.7–109.0; *p* = 0.027) (Figure 8A). Among males, values remained comparable across groups (CL-M: 114.5 ± 14.9 mmHg; 95% CI: 112.3–116.7 vs. CON-M: 113.9 ± 13.4 mmHg; 95% CI: 111.9–115.8; *p* = 0.735). Intraclass analyses confirmed lower SBP in females compared with males across both CL (*p* = 0.001) and CON (*p* = 0.001), consistent with physiological patterns (Figure 8A).

For DBP, CL-F values were comparable to CON-F and to CL-M across all conditions (wakefulness: CL-F: 67.2 ± 2.7 mmHg; 95% CI: 65.3–69.2 vs. CL-M: 71.5 ± 3.8 mmHg; 95% CI: 68.7–74.3; *p* = 0.142; CON-F: 68.1 ± 4.5 mmHg; 95% CI: 64.9–71.4; *p* = 0.589; sleep: CL-F: 55.2 ± 1.7 mmHg vs. CON-F: 54.8 ± 2.6 mmHg; *p* = 0.279; vs. CL-M: 57.7 ± 4.8 mmHg; 95% CI: 52.6–62.8; *p* = 0.359) (Figure 8B).

Nighttime dipping analysis demonstrated that all groups exhibited systolic BP reductions within the reference range (10–20%), indicating a preserved dipping pattern. In the CL group, systolic dipping was 11.7% in females (CL-F) and 12.4% in males (CL-M). In the CON group, values were similar, with 13.0% in females (CON-F) and 12.0% in males (CON-M) (Figure 8A). Diastolic BP dipping also remained within the physiological range, with reductions of 17.9% (CL-F), 19.3% (CL-M), and 19.5% (CON-F). Only CON-M presented an extreme diastolic dipping pattern, with a reduction of 23.2%, exceeding the normal range (Figure 8B). Statistical models confirmed a main effect of time (F = 11.7; *p* < 0.0001), sex (F = 101; *p* < 0.0001), and a significant sex × clinical condition interaction (F = 7.70; *p* = 0.0057) for 24 h SBP variation, while CL alone had no direct effect (*p* = 0.1969), and no time × group interaction was identified (*p* > 0.8087) (Figure 8A).

Within the CL group, SBP in females was significantly lower compared with males at several time points (14 h: 107.1 ± 12.8 vs. 127.9 ± 21.2; *p* = 0.0003; 15 h: 103.2 ± 15.9 vs. 122.3 ± 22.8; *p* = 0.0011; 16 h: 106.1 ± 11.5 vs. 121.0 ± 15.6; *p* = 0.0096; 20 h: 108.3 ± 10.6 vs. 124.4 ± 14.1; *p* = 0.0058; 22 h: 108.5 ± 17.7 vs. 125.6 ± 12.7; *p* = 0.0034; 24 h: 101.4 ± 13.4 vs. 115.5 ± 15.0; *p* = 0.0096) (Figure 7A). No significant differences were found between CON-F and CON-M, or across CON vs. CL within each sex (*p* > 0.0438) (Figure 8A).

For DBP, the 24 h mean confirmed comparable stability across all groups (CL-F: 64.0 ± 12.7 mmHg; 95% CI: 62.2–65.9 vs. CON-F: 64.8 ± 11.9 mmHg; 95% CI: 63.0–66.5; *p* = 0.685; CL-M: 66.7 ± 14.6 vs. CON-M: 65.5 ± 13.3; *p* = 0.573) with no significant intragroup sex differences (CL-F vs. CL-M: *p* = 0.249; CON-F vs. CON-M: *p* = 0.702) (Figure 7B). ANOVA confirmed a main effect of time (F = 17.2; *p* < 0.0001) and sex (F = 6.85; *p* = 0.0091), without group effects (*p* = 0.771). No significant time × sex interactions were identified (*p* = 0.4071). Multiple comparisons revealed point-specific differences: at 15 h (CL-F: 64.3 ± 11.4 vs. CL-M: 77.0 ± 28.7; *p* = 0.0084), 21 h (CON-F: 76.5 ± 16.3 vs. CL-F: 66.3 ± 12.5; *p* = 0.0179), 3 h (CL-F: 54.1 ± 8.0 vs. CL-M: 67.8 ± 15.6; *p* < 0.0084), and between CL-M and CON-M (67.8 ± 15.6 vs. 55.1 ± 8.9; *p* = 0.0084) (Figure 8B).

## 5. Discussion

The present study aimed to characterize hemodynamic, autonomic, vascular, and ambulatory blood pressure responses to maximal exercise in young, physically active adults with a history of LC. We initially hypothesized that LC—particularly among females, who are consistently identified as a higher-risk subgroup for persistent symptoms—would be associated with subtle disturbances in cardiovascular regulation, including an exaggerated pressor response, delayed autonomic recovery, increased arterial stiffness and peripheral resistance, heightened sympathetic dominance, and reduced aerobic capacity. Contrary to these expectations, our findings revealed globally preserved cardiovascular and autonomic integrity across all domains evaluated, with only minor deviations that were small in magnitude and clinically negligible. These results do not support our initial hypothesis and suggest that prior SARS-CoV-2 infection does not meaningfully impair exercise-related cardiovascular regulation in this population. These findings reinforce the relevance of individualized physiological profiling as an essential component of personalized medicine, as preserved responses may guide tailored clinical decisions and exercise-based interventions in young adults with LC.

The BP responses observed throughout CPET and recovery indicate preserved hemodynamic regulation in individuals with LC. The progressive rise in SBP toward maximum effort, accompanied by increased CO, reflects the expected activation of central command, baroreflex resetting, and the exercise pressor reflex, as described in healthy and post-COVID adults [21,22,25]. During recovery, the gradual decline in SBP aligns with post-exercise vasodilation and sympathetic withdrawal, a well-established physiological response following dynamic exercise [46,47]. Although the reductions observed in our study were modest, even small acute decreases in BP—such as reductions of approximately 2–3 mmHg—are associated with meaningful long-term cardiovascular protection [46,48]. Overall, the normal pressor rise, adequate peak responses, and expected recovery trajectory reinforce that BP regulatory mechanisms remain physiologically preserved in young adults with LC, consistent with emerging evidence indicating that cardiorespiratory responses are largely maintained in non-hospitalized LC cohorts [7,22,25].

The HR responses during CPET further support the preservation of autonomic and chronotropic regulation in individuals with LC. All groups reached the expected HRmax for age, with no indication of chronotropic incompetence, a finding consistent with reports showing normal chronotropic responses in non-hospitalized individuals after SARS-CoV-2 infection [21,22]. The rapid decline in HR during the initial minutes of recovery reflects efficient parasympathetic reactivation, a well-established marker of cardiovascular health and autonomic integrity [43,49]. Although LC female group presented slightly lower HR at some recovery time points, the magnitude of these differences was small and not indicative of autonomic impairment. This interpretation aligns with evidence suggesting that autonomic disturbances in LC are heterogeneous and more pronounced in individuals with persistent symptoms or reduced cardiorespiratory fitness [6,7]. In our cohort of physically active young adults, the overall pattern reinforces that LC did not meaningfully disrupt autonomic modulation during or after maximal exercise.

The CO responses observed across groups were consistent with known sex-related differences in cardiovascular physiology. As expected, males demonstrated higher CO values than females, a pattern largely attributed to greater left ventricular mass, larger stroke volume, and higher hemoglobin concentration, features well established in the literature on sex-specific hemodynamic adaptations [20]. Interestingly, this sex-related gap was attenuated in the LC group, suggesting that prior SARS-CoV-2 infection may subtly modulate the typical dimorphic profile without compromising functional capacity. This interpretation aligns with findings reporting that LC can alter autonomic–cardiac interactions, particularly in females, although the magnitude of these effects varies across populations and symptom severity [15,16]. Importantly, all subgroups in our study achieved appropriate CO augmentation during CPET and demonstrated expected early recovery trajectories, consistent with preserved central cardiovascular function in young, physically active adults [21,22]. Together, these observations reinforce that LC did not impair the integrative cardiac adjustments required to support maximal exercise in this cohort. Moreover, these sex-specific physiological patterns reinforce the importance of stratified cardiovascular evaluation within personalized medicine, enabling clinical interpretations that consider individual variability and support more tailored exercise and follow-up strategies.

The behavior of TPR and PVR across groups further supports the preservation of peripheral vascular regulation in individuals with LC. As expected, females in the CON group exhibited transiently higher vascular resistance during early recovery, a pattern consistent with known sex-related differences in arterial diameter, vasoconstrictor responsiveness, and microvascular tone [20]. Notably, this pattern was not present in LC female group, suggesting that prior SARS-CoV-2 infection may subtly influence the typical dimorphic response without producing overt dysfunction. From a physiological standpoint, vascular resistance during and after exercise reflects the dynamic balance between metabolic vasodilation in active muscle and sympathetic vasoconstriction in non-active vascular beds, an interaction that remains robust in healthy adults [47,48]. The absence of exaggerated vasoconstriction or impaired recovery in LC groups aligns with recent findings indicating preserved peripheral vascular function in non-hospitalized individuals with mild or moderate LC symptoms [7,13]. Taken together, the present results reinforce that LC did not adversely affect the peripheral vascular adjustments required to sustain and recover from maximal exertion in this physically active cohort.

The vascular indices evaluated in this study, including PWV and AIx75, also indicated preserved central vascular function in individuals with LC. In the control group, PWV was higher in males than in females, a difference consistent with known sex-related variations in arterial compliance and the modulatory role of estrogens on vascular stiffness [20]. Interestingly, this sex-related difference was not observed among participants in the LC group, suggesting that prior SARS-CoV-2 infection may subtly modulate typical dimorphic patterns without leading to overt vascular impairment. AIx75 tended to be lower in female participants with LC compared with female controls, a pattern that coincided with slightly lower HR during recovery, suggesting efficient autonomic–vascular coupling rather than a maladaptive response. These findings contrast with reports of persistent arterial stiffness or endothelial dysfunction in some LC cohorts [11,12,14], but align with emerging evidence showing substantial heterogeneity across post-COVID phenotypes, particularly in young, physically active adults with mild or moderate acute infection [13]. Overall, our results reinforce that LC did not meaningfully disrupt central vascular dynamics in this population.

The HRV findings further support the preservation of autonomic function in individuals with LC. Female participants with LC demonstrated higher HF values at rest and during some recovery time points, indicating robust parasympathetic modulation, whereas male participants tended to return to baseline values more rapidly. This pattern is consistent with known sex-related differences in autonomic regulation, where females often exhibit greater vagal predominance and slower autonomic recovery [20]. Importantly, no subgroup demonstrated signs of sustained sympathetic overactivity, reduced parasympathetic reactivation, or altered LF/HF balance, findings that contrast with reports describing dysautonomia, impaired HRV, and autonomic disturbances in some LC populations [7,10,48]. The heterogeneity observed across studies may reflect differences in symptom burden, physical activity status, and severity of the initial infection. In the present sample composed of young, physically active adults, the overall pattern suggests that LC did not compromise sympathovagal balance during or after maximal exercise. These results align with emerging evidence indicating that individuals with higher fitness levels exhibit better preserved autonomic responses following SARS-CoV-2 infection [7].

Assessment of ambulatory BP over 24 h after maximal effort revealed no clinically relevant changes in pressure modulation. Analysis of systolic dipping revealed reductions within the expected physiological range (10–20%) in all groups, indicating preservation of circadian modulation of blood pressure [42,50,51]. This behavior reinforces that, in young, normotensive and physically active adults, previous SARS-CoV-2 infection did not compromise the expected circadian pattern of BP. DBP behavior also remained physiological in most subgroups, with the exception of the male CON group (CON-M), which showed extreme dipping (23.2%), a characteristic described in the literature as a potential risk factor in hypertensive elderly people, but which in young and active populations tends to reflect greater autonomic and vascular efficiency, without immediate clinical implications [50,51,52]. Previous studies in normotensive young people have also shown that dipping within the 10–20% range is common and associated with adequate autonomic response between wakefulness and sleep [53,54]. Therefore, the absence of reduced dipping in our sample reinforces the integrity of autonomic regulation and pressure dynamics, highlighting that individual variations such as extreme dipping should be interpreted in the context of demographics, behavior and level of physical activity, rather than as an isolated pathological marker.

The strengths of this study include rigorous methodological procedures, appropriate sample size calculation, stratification by biological sex, and homogeneity between groups in terms of body composition, physical activity level, vaccination status, and time since infection. These aspects enhance internal validity and minimize confounding. Limitations of this study should be acknowledged. First, the absence of biochemical and endothelial biomarkers restricted mechanistic interpretation, and the reliance on symptom-based definitions of LC may have introduced heterogeneity in case classification. Retrospective symptom reporting also depended on participant recall, which may introduce recall bias and affect the accuracy of symptom frequency estimates. The lack of a never-infected control group and the restriction to young, healthy, physically active adults limit external generalizability and prevent direct extrapolation to older or comorbid populations. From a methodological standpoint, arterial stiffness and central hemodynamic indices were assessed using an oscillometric device. Although carotid–femoral tonometry remains the gold standard for PWV, oscillometric methods are widely accepted due to their practicality, reproducibility, and strong correlation with reference techniques. Nonetheless, these devices estimate central pressures indirectly through mathematical models and transfer functions, whose accuracy depends on device calibration, individual arterial properties, and algorithmic assumptions. Such factors may introduce variability and limit the precision of absolute central BP values and derived indices, and their validity may differ across populations and clinical conditions.

Future research should build on these findings by incorporating more comprehensive and mechanistic assessments capable of capturing subtle cardiovascular alterations that may not be detectable through oscillometric or exercise-based measurements alone. Multimodal vascular evaluations—including carotid–femoral PWV via applanation tonometry, endothelial function testing such as flow-mediated dilation, and microvascular reactivity analyses—would allow a more granular characterization of vascular health in individuals recovering from SARS-CoV-2 infection [12,14]. Additionally, longitudinal designs are needed to determine whether preserved responses observed in young, physically active adults persist over time or differ in those with varying symptom burden, fitness levels, or comorbidities. Expanding the sample to include older adults, sedentary individuals, or patients with persistent exertional intolerance may help elucidate heterogeneous cardiovascular trajectories reported in LC cohorts [26]. Integrating immunological, autonomic, and vascular biomarkers would further clarify potential mechanistic pathways linking SARS-CoV-2 infection to exercise-related physiological responses. Collectively, such approaches will strengthen the understanding of LC phenotypes and improve the identification of individuals who may benefit from tailored rehabilitation and exercise strategies.

## 6. Conclusions

In conclusion, Long COVID was not found to be associated with clinically meaningful impairments in hemodynamic, autonomic, or vascular responses to maximal exercise in young physically active adults. In females, despite greater acute symptom burden and a shorter infection-to-assessment interval, functional performance and cardiovascular recovery were preserved. By revealing sex-specific yet functionally preserved physiological profiles, our results contribute to personalized medicine by supporting individualized cardiovascular assessment and more tailored exercise guidance for young adults recovering from LC.

## Figures and Tables

**Table 1:** Baseline characteristics: anthropometry and body composition.

	Long COVID (*n* = 18)		Control (*n* = 20)		* *p* Value			
	Female (*n* = 10)	Male (*n* = 8)	Female (*n* = 10)	Male (*n* = 10)	* *p* Value (Between-Group Female)	* *p* Value (Between-Group Male)	# *p* Value (Within-Group LC)	# *p* Value (Within-Group CON)
***Anthropometric parameters***								
Age (years)	28.1 ± 7.5 (22.7–33.5)	28.0 ± 8.0 (21.3–34.7)	22.9 ± 3.2 (20.6–25.2)	27.1 ± 6.7 (22.3–31.9)	0.066	0.803	0.892	0.308
Height (cm)	160.9 ± 6.2 (156.4–165.4) ^#^	180.6 ± 7.9 (174.0–187.2)	164.1 ± 5.7 (159.9–168.2) ^#^	175.8 ± 8.7 (169.5–182.0)	0.248	0.236	<0.01	0.004
Body mass (kg)	64.7 ± 6.3 (60.2–69.2) ^#^	88.3 ± 12.0 (78.3–98.3)	66.8 ± 13.1 (57.4–76.2)	76.4 ± 14.7 (65.9–86.9)	0.654	0.076	<0.01	0.264
Neck circumference (cm)	33.1 ± 2.3 (31.4–34.7) ^#^	38.1 ± 1.8 (36.6–39.6)	32.1 ± 2.6 (30.2–33.9) ^#^	38.8 ± 3.9 (36.1–41.6)	0.389	0.634	<0.01	0.006
Waist circumference (cm)	74.4 ± 5.7 (70.4–78.5) ^#^	87.4 ± 8.2 (80.6–94.3)	73.7 ± 7.5 (68.4–79.1)	82.5 ± 9.9 (75.4–89.6)	0.814	0.266	0.002	0.116
Abdominal circumference (cm)	83.9 ± 6.9 (78.9–88.8) ^#^	94.7 ± 9.2 (87.1–102.4)	83.7 ± 10.3 (76.4–91.1)	87.5 ± 10.3 (80.1–94.8)	0.974	0.132	0.011	0.750
Hip circumference (cm)	101.2 ± 6.5 (96.5–105.8)	103.6 ± 5.9 (98.7–108.6)	101.7 ± 5.6 (97.7–105.7)	99.5 ± 8.7 (93.2–105.7)	0.852	0.253	0.265	0.400
Waist–hip ratio	0.81 ± 0.1 (0.7–0.9)	0.9 ± 0.1 (0.8–0.9)	0.9 ± 0.1 (0.8–0.9)	0.8 ± 0.1 (0.8–0.9)	0.339	0.072	0.055	0.107
Waist–height ratio	0.5 ± 0.04 (0.4–0.5)	0.5 ± 0.1 (0.4–0.5)	0.5 ± 0.04 (0.4–0.5)	0.5 ± 0.04 (0.4–0.5)	0.414	0.488	0.328	0.728
***Body composition parameters***								
Body mass index (kg/m^2^)	24.4 ± 1.9 (23.0–25.9) ^#^	27.3 ± 2.9 (24.8–29.7)	24.8 ± 3.7 (22.1–27.4)	24.9 ± 2.8 (22.8–26.9)	0.810	0.097	0.028	0.706
Lean body mass (kg)	23.5 ± 2.1 (21.8–25.1) ^#^	39 ± 4.9 (34.9 –4 3.1)	24.8 ± 3.2 (22.5–27.1) ^#^	36.0 ± 6.8 (31.2–40.9)	0.292	0.301	<0.01	0.001
Fat body mass (kg)	20.1 ± 2.6 (18.1–22.1)	20.1 ± 6.0 (15.1–25.2) *	22.0 ± 8.8 (15.7–28.3) ^#^	13.3 ± 4.6 (10.0–16.6)	0.521	0.02	0.974	0.010
Body fat (%)	32.0 ± 0.9 (27.4–36.8) ^#^	22.5 ± 1.7 (12.8–29.2) *	31.9 ± 2.1 (22.8–43.6) ^#^	17.2 ± 1.1 (11.8–23)	0.971	0.015	<0.01	<0.01
Visceral fat area (cm^2^)	79.9 ± 11.8 (70.9–88.9)	90.9 ± 28.9 (66.7– 115.1) *	85.7 ± 31.6 (63.1–108.3) ^#^	58.2 ± 24.4 (40.7–75.6)	0.598	0.023	0.312	0.029

Values are presented as mean ± standard deviation and confidence interval; between-group analysis: female control vs. female Long COVID. and male control vs. male Long COVID; within-group analysis: female control vs. male control. and female Long COVID vs. male Long COVID; * indicates significant differences between groups of the same sex (LC vs. CON) (*p* < 0.05); # indicates significant within-group sex differences (*p* < 0.05). Regarding body composition, female participants presented a higher body fat percentage (CL-F: 32.0 ± 0.9%; CL-M: 22.5 ± 1.7%; *p* = 0.0001), whereas males displayed greater lean mass (CL-F: 23.5 ± 2.1 kg; CL-M: 39.0 ± 4.9 kg; *p* = 0.0000003) (Table 1). Visceral adipose tissue was significantly higher in CL-M participants (90.9 ± 28.9 cm^2^) compared with CON-M (58.2 ± 24.4 cm^2^; *p* = 0.023). There were no significant differences among females (CL-F: 79.9 ± 11.8 cm^2^; CON-F: 85.7 ± 31.6 cm^2^; *p* = 0.598) (Table 1).

**Table 2:** The table presents the distribution of hormonal contraceptive methods used by female participants and the number of children (offspring) in two groups: participants with Long COVID and the control group.

Female Sex Participants	Long COVID (*n* = 10)	Control (*n* = 10)
***Contraceptive method***		
None	3 (30%)	2 (20%)
Oral	7 (70%)	5 (50%)
Subdermal	0 (0%)	1 (10%)
Intrauterine device	0 (0%)	1 (10%)
Unspecified	0 (0%)	1 (10%)
***Offspring***		
1	1 (10%)	0 (0%)

Data are expressed as frequency (percentage).

**Table 3:** Baseline characteristics of the study population, including acute phase severity, infection history, vaccination and symptoms (acute and persistent).

	Long COVID (*n* = 18)		Control (*n* = 20)		* *p* Value			
	Female (*n* = 10)	Male (*n* = 8)	Female (*n* = 10)	Male (*n* = 10)	* *p* Value (Between-Group Female)	* *p* Value (Between-Group Male)	# *p* Value (Within-Group LC)	# *p* Value (Within-Group CON)
***Acute Phase Severity Classification***								
Mild	2 (20%)	6 (75%)	6 (60%)	7 (70%)	–	–	–	–
Moderate	8 (80%)	2 (25%)	4 (40%)	3 (30%)	–	–	–	–
***Number of positive diagnoses for COVID-19***								
1	7 (70%)	5 (63%)	7 (70%)	8 (80%)	–	–	–	–
2	3 (30%)	2 (25%)	3 (30%)	1 (10%)	–	–	–	–
3	0	0	0	1 (10%)	–	–	–	–
4	0	1 (12%)	0	0	–	–	–	–
***Time between last diagnosis and CPET***								
Window between last infection (positive test) and sample collection [months]	14.8 ± 4.11 (2–35) *	26.88 ± 5.14 (2–47)	27.0 ± 4.11 (2–49)	28.5 ± 3.02 (14–46)	0.049	0.778	0.081	0.475
Window between last infection (positive test) and sample collection [days]	444 ± 123.2 (60–1050) *	806 ± 154.2 (60–1410)	810 ± 123.4 (60–1470)	855 ± 90.7 (420–1380)	0.049	0.778	0.081	0.475
***Types and doses of vaccines***								
Coronavac (0)	5 (50%)	6 (75%)	5 (50%)	7 (70%)	–	–	–	–
Coronavac (1)	2 (20%)	1 (12%)	1 (10%)	1 (10%)	–	–	–	–
Coronavac (2)	3 (30%)	1 (12%)	4 (40%)	2 (20%)	–	–	–	–
Sinovac (0)	10 (100%)	7 (87%)	10 (100%)	9 (90%)	–	–	–	–
Sinovac (1)	0	1 (12.5%)	0	1 (10%)	–	–	–	–
Astrazeneca (0)	6 (60%)	5 (62%)	7 (70%)	8 (80%)	–	–	–	–
Astrazeneca (1)	2 (20%)	1 (12%)	1 (10%)	1 (10%)	–	–	–	–
Astrazeneca (2)	2 (20%)	2 (25%)	2 (20%)	2 (20%)	–	–	–	–
Pfizer (0)	1 (10%)	3 (37%)	2 (20%)	2 (20%)	–	–	–	–
Pfizer (1)	2 (20%)	1 (12%)	1 (10%)	2 (20%)	–	–	–	–
Pfizer (2)	4 (40%)	3 (37%)	4 (40%)	2 (20%)	–	–	–	–
Pfizer (3)	3 (30%)	1 (12%)	2 (20%)	1 (10%)	–	–	–	–
Pfizer (4)	0	0	0	3 (30%)	–	–	–	–
Pfizer (5)	0	0	1 (10%)	0	–	–	–	–
Jansen (0)	10 (100%)	5 (62%)	8 (80%)	9 (90%)	–	–	–	–
Jansen (1)	0	3 (37%)	2 (20%)	1 (10%)	–	–	–	–
***Symptoms in the acute phase of COVID-19***								
Throat irritation	0	0	1 (10%)	0	–	–	–	–
Nasal congestion	10 (100%)	1 (12%)	2 (20%)	1 (10%)	–	–	–	–
Headache/Migraine	1 (10%)	6 (75%)	4 (40%)	5 (50%)	–	–	–	–
Throat sore	2 (20%)	1 (12%)	2 (20%)	0	–	–	–	–
Myalgia	2 (20%)	1 (12%)	2 (20%)	1 (10%)	–	–	–	–
Abdominal pain	0	1 (12%)	0	0	–	–	–	–
Fatigue	10 (100%)	6 (75%)	7 (70%)	4 (40%)	–	–	–	–
Shortness of breath	4 (40%)	2 (25%)	0	1 (10%)	–	–	–	–
Fever	10 (100%)	6 (75%)	5 (50%)	5 (50%)	–	–	–	–
Memory loss	0	1 (12%)	0	0	–	–	–	–
Difficulty concentrating	0	0	0	0	–	–	–	–
Anosmia	1 (10%)	2 (25%)	0	1 (10%)	–	–	–	–
Ageusia	1 (10%)	3 (37%)	0	1 (10%)	–	–	–	–
Dizziness	0	1 (12%)	0	0	–	–	–	–
Loss of appetite	0	1 (12%)	0	0	–	–	–	–
Cough	10 (100%)	5 (62%)	8 (80%)	6 (60%)	–	–	–	–
Runny nose	0	0	0	0	–	–	–	–
Hair loss	1 (10%)	0	0	0	–	–	–	–
Number of symptoms in the acute phase	5.1 ± 0.31 (4–7) *	4.5 ± 0.8 (2–9) *	3.27 ± 0.43 (1–6)	2.5 ± 0.5 (0–5)	0.003	0.042	0.081	0.398
***Persistent Long COVID Symptoms***								
Fatigue	2 (20%)	2 (25%)	0	0	–	–	–	–
Low immunity	1 (10%)	0	0	0	–	–	–	–
Anosmia	1 (10%)	1 (12%)	0	0	–	–	–	–
Ageusia	1 (10%)	1 (12%)	0	0	–	–	–	–
Cough	1 (10%)	0	0	0	–	–	–	–
Memory loss	1 (10%)	3 (37%)	0	0	–	–	–	–
Abdominal pain	0	1 (12%)	0	0	–	–	–	–
Hair loss	2 (20%)	0	0	0	–	–	–	–
Sore throat	1 (10%)	0	0	0	–	–	–	–
Headache	0	1 (12%)	0	0	–	–	–	–
Difficulty concentrating	1 (10%)	0	0	0	–	–	–	–
Chest pain	0	1 (12%)	0	0	–	–	–	–
Sinusitis	1 (10%)	0	0	0	–	–	–	–
Number of persistent symptoms	1.3 ± 0.2 (1–3)	1.4 ± 0.2 (1–2)	0 ± 0 (0–0)	0 ± 0 (0–0)	0	0	0	0

Values are presented as mean ± standard deviation and confidence interval; between-group analysis: female control vs. female Long COVID. and male control vs. male Long COVID; within-group analysis: female control vs. male control. and female Long COVID vs. male Long COVID; * indicates significant differences between groups of the same sex (LC vs. CON) (*p* < 0.05); # indicates significant within-group sex differences (*p* < 0.05). – indicates that no statistical test was performed for these categorical frequencies.

**Table 4:** Characterization of the cardiopulmonary exercise test (CPET), cardiorespiratory fitness, biochemical parameters, cardiovascular responses and performance.

	Long COVID (*n* = 18)		Control (*n* = 20)		* *p* Value			
	Female (*n* = 10)	Male (*n* = 8)	Female (*n* = 10)	Male (*n* = 10)	* *p* Value (Between-Group Female)	* *p* Value (Between-Group Male)	# *p* Value (Within-Group LC)	# *p* Value (Within-Group CON)
***Cardiorespiratory fitness and physical activity levels***								
peak VO_2_ (mL/Kg.min)	36.4 ± 7.1 (31.8–41.0)	42.5 ± 9.3 (36.0–48.9)	37.7 ± 7.5 (33.1–42.4) #	45.9 ± 5.6 (42.5–49.4)	0.696	0.328	0.162	0.013
***Biochemical parameters (at rest)***								
Blood glucose (mg.dL)	94.4 ± 10.7 (86.7–102.1)	92.7 ± 10.7 (83.8–101.7)	93.8 ± 9.5 (86.4–101.1)	85.7 ± 9.2 (78.1–93.4)	0.894	0.182	0.749	0.097
***Number of complete stages during CPET***								
3	4 (40%)	1 (12.5%)	2 (20%)	0	–	–	–	–
4	5 (50%)	5 (62.5%)	6 (60%)	5 (50%)	–	–	–	–
5	1 (10%)	2 (25%)	2 (20%)	3 (30%)	–	–	–	–
6	0	0	0	2 (20%)	–	–	–	–
Duration of CPET (seg)	687.1 ± 99.2 (625.6–748.6)	716.8 ± 131.6 (625.6–808.11)	686.9 ± 128.0 (607.5–766.24) #	810.1 ± 133.4 (727.3–892.8)	0.996	0.158	0.604	0.049
Estimated maximum HR (Tanaka equation)	188.3 ± 5.2 (185.1–191.5)	188.5 ± 5.6 (184.6–192.4)	192.0 ± 2.2 (190.6–193.3)	188.9 ± 4.7 (185.9–191.8)	0.084	0.821	0.815	0.071
Prediction of 90% HR based on the Tanaka equation	169.5 ± 4.6 (166.6–172.3)	169.6 ± 4.9 (166.1 –173.0)	172.8 ± 2.2 (171.4–174.1)	170.1 ± 4.2 (167.4–172.7)	0.085	0.892	1	0.109
Maximum HR achieved during CPET	182.0 ± 9.8 (175.95–188.0)	187.9 ± 5.2 (184.2–191.5)	188.9 ± 7.1 (184.4–193.3)	184.9 ± 10.3 (178.5–191.3)	0.057	0.755	0.124	0.324
% achieved HR/predicted HR	96.7 ± 4.2 (94.1–99.2)	99.7 ± 2.6 (97.9–101.5)	98.4 ± 3.4 (96.3–100.5)	98.0 ± 4.4 (95.3–100.7)	0.285	0.334	0.076	0.730
Achieved more than 90% of expected HR	10 (100%)	8 (100%)	10 (100%)	9 (90%)	–	–	–	–
Maximum Respiratory Exchange Ratio	1.1 ± 0.1 (1.0–1.2)	1.1 ± 0.1 (1.1–1.2)	1.1 ± 0.1 (1.1–1.2)	1.1 ± 0.1 (1.0–1.1)	0.819	0.343	0.832	0.338
Voluntary interruption	10 (100%)	8 (100%)	10 (100%)	10 (100%)	–	–	–	–
max BORG > 17	6 (60%)	5 (62%)	4 (40%)	7 (70%)	–	–	–	–
max BORG < 17	4 (40%)	3 (37%)	6 (60%)	3 (30%)	–	–	–	–
Maximum test parameters achieved	10 (100%)	7 (87%)	6 (60%)	10 (100%)	–	–	–	–
***VO2 Peak Classification***								
Very Poor	0	0	0	0	–	–	–	–
Weak	2 (20%)	0	2 (20%)	0	–	–	–	–
Regular	2 (20%)	4 (50%)	3 (30%)	2 (20%)	–	–	–	–
Good	5 (50%)	2 (25%)	5 (50%)	6 (60%)	–	–	–	–
Excellent	0	2 (25%)	0	2 (20%)	–	–	–	–
Rate-pressure product at rest	6663.8 ± 1277.5 (5871.9–7455.6)	7072.3 ± 1671.4 (5914.0–8230.6)	7984.7 ± 648.2 (7582.9–8386.4)	7134.4 ± 1183.3 (6400.9–7867.8)	0.835	0.9277	0.564	0.061
Peak rate-pressure product	25,177.1 ± 4865.6 (22,161.3–28,192.8) #	30,309.7 ± 3354.0 (27,985.5–32,633.9)	27,055.9 ± 3687.1 (24,770.5–29,341.2) #	31,759.5 ± 3446.5 (29,623.2–33,895.7)	0.343	0.382	0.022	0.008
***IPAQ***								
IPAQ—Sedentary	0	0	0	0	–	–	–	–
IPAQ—Irregularly active A	0	0	0	0	–	–	–	–
IPAQ—Irregularly active B	1 (10%)	0	1 (10%)	0	–	–	–	–
IPAQ—Active	9 (90%)	7 (100%)	9 (90%)	10 (100%)	–	–	–	–
IPAQ— Very active	0	0	0	0	–	–	–	–

Values are presented as mean ± standard deviation and confidence interval; between-group analysis: female control vs. female Long COVID. and male control vs. male Long COVID; within-group analysis: female control vs. male control. and female Long COVID vs. male Long COVID; * indicates significant differences between groups of the same sex (LC vs. CON) (*p* < 0.05); # indicates significant within-group sex differences (*p* < 0.05). – indicates that no statistical test was performed for these categorical frequencies. Resting glycemia was homogeneous across groups: CL-F (94.4 ± 10.7 mg/dL) vs. CON-F (93.8 ± 9.5 mg/dL; *p* = 0.894), and CL-M (92.7 ± 10.7 mg/dL) vs. CON-M (85.7 ± 9.2 mg/dL; *p* = 0.182), with no significant sex-related differences within the control group despite numerically lower values in males (*p* = 0.097) (Table 4).

## Data Availability

The original contributions presented in this study are included in the article. Further inquiries can be directed to the corresponding author.

## Abbreviations

LC	Long COVID
CON	Control
LCF	Long COVID–female
LCM	Long COVID–male
CONF	Control–female
CONM	Control–male
PREs	Pre-exercise seated
PREp	Pre-exercise standing
MAX	Maximum effort
IA	Immediately after exercise
A3min	3 minutes after exercise
A5min	5 minutes after exercise
A10min	10 minutes after exercise
A30min	30 minutes after exercise
A60min	60 minutes after exercise
A24h	24 hours after CPET
CPET	Cardiopulmonary Exercise Testing
ABPM	Ambulatory Blood Pressure Monitoring
PARQ	Physical Activity Readiness Questionnaire
IPAQ	International Physical Activity Questionnaire
VO2 peak	Peak oxygen uptake
HRmax	Predicted maximal heart rate
InBody770	Multifrequency bioimpedance analyzer
VO2000	Gas analyzer
PolarH10	Heart rate transmitter
SBP	Systolic blood pressure
DBP	Diastolic blood pressure
BP	Blood pressure
HR	Heart rate
CO	Cardiac output
RPP	Rate–pressure product
TPR	Total vascular resistance
PVR	Peripheral vascular resistance
PWV	Pulse wave velocity
AIx	Augmentation index
AIx75	Augmentation index standardized to 75 bpm
HRV	Heart rate variability
HF	High-frequency component
LF	Low-frequency component
LFHF	LF/HF ratio
